# krCRISPR: an easy and efficient strategy for generating conditional knockout of essential genes in cells

**DOI:** 10.1186/s13036-019-0150-y

**Published:** 2019-04-24

**Authors:** Bei Wang, Zishi Wang, Daqi Wang, Baolong Zhang, Sang-Ging Ong, Mingqing Li, Wenqiang Yu, Yongming Wang

**Affiliations:** 1 MOE Key Laboratory of Contemporary Anthropology at School of Life Sciences and Zhongshan Hospital, Fudan University, Shanghai, 200438 China; 2 Shanghai Public Health Clinical Center & Laboratory of RNA Epigenetics, Institute of Biomedical Sciences, Shanghai Medical College, Fudan University, Shanghai, 201508 China; 3 Department of Pharmacology, University of Illinois College of Medicine, Chicago, IL 60612 USA; 4 Division of Cardiology, Department of Medicine, University of Illinois College of Medicine, Chicago, IL 60612 USA; 5 The Key Lab of Reproduction Regulation of NPFPC in SIPPR, Institute of Reproduction & Development in Obstetrics & Gynecology Hospital, Fudan University, Shanghai, 200011 China

**Keywords:** Episomal vector, CRISPR/Cas9, Essential gene, Knockout

## Abstract

**Background:**

CRISPR/Cas9 system is a powerful tool for knocking out genes in cells. However, genes essential for cell survival cannot be directly knocked out. Traditionally, generation of conditional knockout cells requires multiple steps.

**Results:**

In this study, we developed an easy and efficient strategy to generate conditional knockout cells by using double episomal vectors – one which expresses gRNA and Cas9 nuclease, and the other expresses an inducible rescue gene. Using this system which we named “krCRISPR” (knockout-rescue CRISPR), we showed that essential genes, *HDAC3* and *DNMT1*, can be efficiently knocked out. When cells reach a desired confluency, the exogenous rescue genes can be silenced by the addition of doxycycline. Furthermore, the krCRISPR system enabled us to study the effects of the essential gene mutations on cells. We showed that the P507L mutation in *DNMT1* led to downregulation of global DNA methylation in cells, indicating that it is a disease-causing mutation.

**Conclusions:**

The krCRISPR system offers an easy and efficient platform that facilitates the study of essential genes’ function.

**Electronic supplementary material:**

The online version of this article (10.1186/s13036-019-0150-y) contains supplementary material, which is available to authorized users.

## Background

Deletion of a target gene in cells and observation of the resulting phenotype is a common strategy to determine the function of a gene in biological research [1–3]. However, numerous genes that are essential for cell viability result in cell death when they are ablated [3–5]. Recent genome-wide screening has revealed that essential genes account for ~ 10% of total human genes [6]. To study essential genes in cells, conditional knockout strategies have been developed.

The Cre/loxP recombination system is the most commonly used technique to knockout essential genes. This technique requires insertion of a pair of 34 bp loxP sites flanking the target genes, and expression of Cre recombinase enzyme will cause a deletion of the genes between the two loxP sites [7, 8] (Additional file 1: Figure S1A). Other conditional strategies have also been developed. Liao et al. generated a transgenic cell line that expresses an essential gene under the control of P_tet_ promoter (Doxycycline-inducible gene expression), and then knocked out the endogenous target gene [3]. Upon desirable cell density, the expression of the exogenous gene was shut down [3] (Additional file 1: Figure S1B). Matsunaga et al. inserted a tetracycline-regulated inducible gene promoter (tet-OFF/TRE-CMV) upstream of the endogenous target gene in cells that express tetracycline transactivator (tTA) [9]. The inserted promoter disrupted the endogenous promoter and controlled endogenous gene expression by doxycycline (Dox) [9] (Additional file 1: Figure S1C). Nevertheless, both strategies required multiple steps to generate stable integration cell lines. The combination of the CRISPR/Cas9 and episomal vector technology represent an alternative strategy to knockout essential genes.

The RNA-guided CRISPR/Cas9 system is a powerful tool for genome editing in diverse organisms and cell types [10–12]. CRISPR/Cas9 system consists of two components: a Cas9 nuclease and a 100 nucleotide guide RNA (gRNA) which directs Cas9 to cleave the target sites and generate double-strand breaks (DSBs) [13]. As an RNA-guided DNA endonuclease, Cas9 can be easily programmed to target new sites by altering its gRNA sequence. The DSBs can be repaired by the cell’s endogenous DNA repair machinery through homology-directed repair (HDR) using an introduced DNA repair template, such as a double-stranded DNA donor plasmid or a single-stranded oligo DNA nucleotide (ssODN), enabling knock-in of precise mutations or reporters [14–16]. The DSBs can also be repaired by non-homologous end-joining (NHEJ), resulting in nonspecific small insertions and deletions (indels) useful for generating loss-of-function mutations [1, 3].

We have previously used an episomal vector to express gRNA and Cas9 nuclease, and achieved high efficiency of gene knockout [17]. Episomal vector allows for long-term genome editing with the puromycin resistance gene on the episomal vector enabling enrichment of transfected cells. In this study, we developed an easy and efficient strategy to generate gene knockout-rescue systems with two episomal vectors, allowing conditional knockout of essential genes. Using this system, one plasmid encodes Cas9 and gRNA for essential gene knockout, and the other plasmid encodes the target gene controlled by Tet-Off system. This system is designated as krCRISPR (knockout-rescue CRISPR). We showed two examples of essential gene knockout using this system. We further showed that the effects of specific mutation can be studied by replacing wild-type (WT) essential gene with mutant versions. Our system will facilitate functional studies of essential genes.

## Results

### Establishment of a knockout-rescue system by using double episomal vectors

**Fig. 1:**
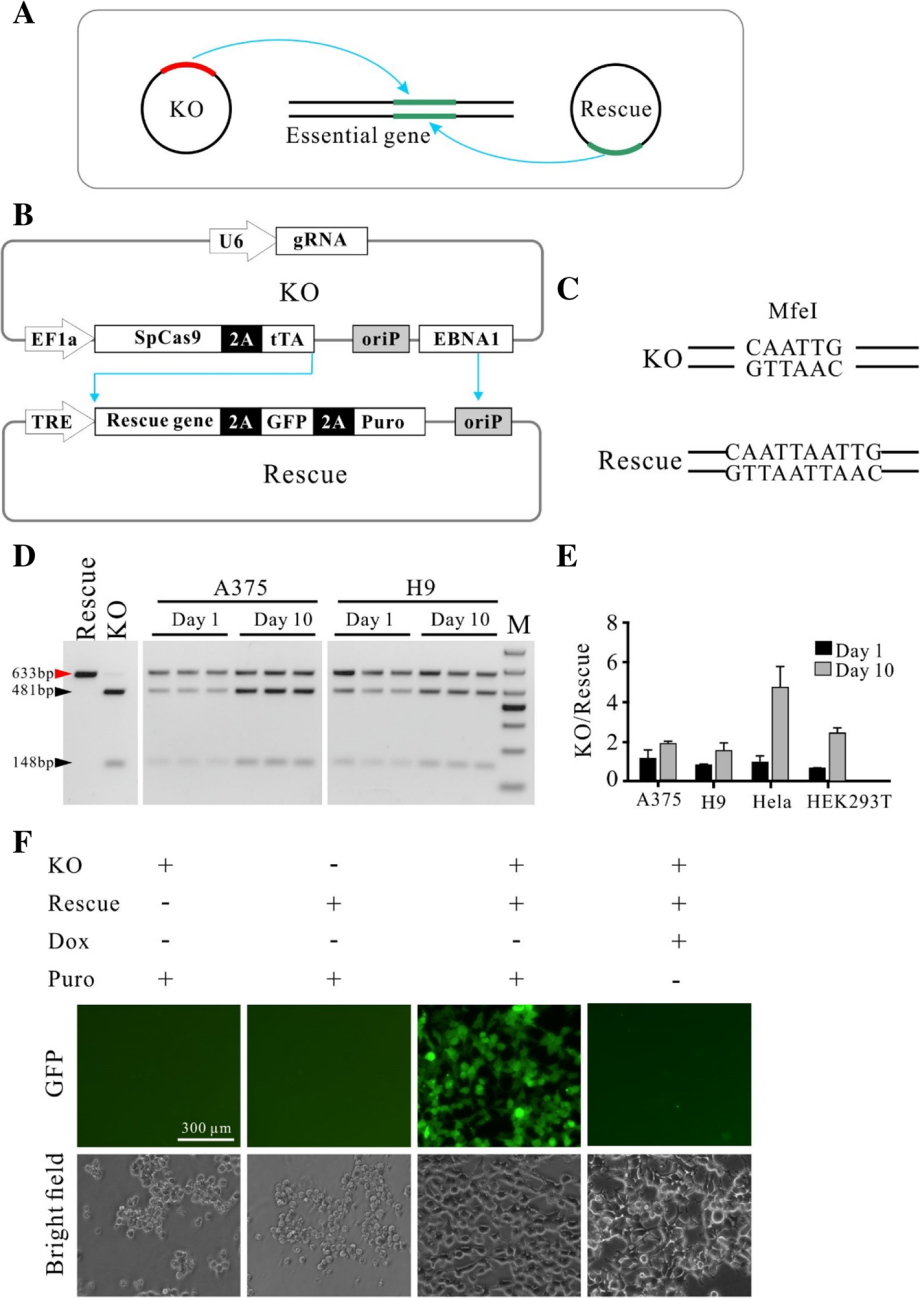
Establishment of the knockout-rescue system with double episomal vectors. **a** Schematic of the krCRISPR system design. This system consists of two plasmids, KO and Rescue. KO plasmid is for gene knockout, and Rescue plasmid is for gene rescue. **b** Schematic of the plasmid design. KO plasmid contains a hU6 promoter-driven gRNA and an EF1α promoter-driven Cas9 nuclease for gene knockout, a tTA gene for inducible gene expression, and OriP/EBNA1 elements for the episomal maintenance of the plasmid in the cells. Cas9 and the tTA gene used the same promoter but were separated by a P2A peptide. Rescue plasmid contains a TRE promoter-driven rescue gene, puromycin resistance gene and copGFP separated with P2A peptides, and an OriP element for the plasmid replication. **c** An MfeI restriction site was removed from the Rescue plasmid by digestion and religation. **d** Representative gel pictures of RFLP analysis of double plasmids at day 1 and day 10 after transfection in A375 and H9 cells. KO and Rescue plasmids contain a common region that can be amplified by a pair of primers. An MfeI restriction site is only present on the KO plasmid and digestion of the PCR products resulted in two bands (481 + 148 bp). A 633 bp fragment amplified from Rescue plasmid could not be digested by MfeI. Two lanes on the left are the PCR products amplified from plasmid DNA and used as a control. Each lane on the middle and right gels presented an independent transfection. **e** The ratio of the two plasmids was quantified based on RFLP analysis (*n* = 3, error bars showed mean ± SEM). **f** Representative images of the cells transfected with single or double plasmids. Cells transfected with single plasmid could not survive with puromycin selection, while cells transfected with double plasmids could survive and express GFP. GFP expression was inhibited by addition of Dox for 3 days

In order to knockout essential genes in cells, we designed a gene knockout-rescue system with double episomal vectors (Fig. 1a). Double vectors can accommodate multiple genetic components. One plasmid which encodes Cas9 and gRNA for gene knockout was designated as KO (knockout) plasmid and the other plasmid which encodes the rescue gene was designated as Rescue plasmid. The advantage of the episomal vectors is that they can replicate in eukaryotic cells, allowing long-term Cas9 and gRNA expression [17]. The episomal vector used in this study was derived from Epstein-Barr virus (EBV) which contains two components essential for the episomal maintenance in cells: the latent origin oriP and its binding protein Epstein-Barr-associated nuclear antigen 1 (EBNA1) [18]. In order to simultaneously retain two episomal plasmids in cells, the EBNA1 coding sequence was removed from the Rescue plasmid, resulting in the episomal maintenance of the Rescue plasmid solely dependent on the KO plasmid (Fig. 1b). The Rescue plasmid encodes three genes: rescue gene, GFP and puromycin resistant gene (Puro), separated by self-cleaving T2A peptide. All three genes’ expression was controlled by the Tet-Off system. In order to reduce leaky expression, the tTA gene was encoded by the KO plasmid. Under puromycin selection, cells’ survival depends on the Rescue plasmid that expresses puromycin resistant gene. Therefore, cells’ survival requires the episomal maintenance of both plasmids.

We first tested whether both plasmids can be simultaneously maintained in cells. Both plasmids contain a common sequence that has an MfeI restriction site. To differentiate between these plasmids by restriction fragment length polymorphism (RFLP) assay, the MfeI restriction site on the Rescue plasmid was destroyed by digestion and religation (Fig. 1c). We co-transfected the same amount of each plasmid into different cell types and PCR-amplified the common region for the RFLP assay. Twenty-four hours post-transfection, a similar amount of both plasmids was detected (Fig. 1d, e and Additional file 1: Figure S2A). After 10 days of puromycin selection, higher amount of the KO plasmid was detected. A possible reason is that the KO plasmid retained the intact oriP/EBNA1 sequence which favors plasmid replication. To rule out the possibility that only one plasmid was present in a portion of cells, we transfected individual plasmid into cells and selected with puromycin for three days. As expected, neither KO plasmid nor Rescue plasmid could support cell’s survival (Fig. 1f). Co-transfection of the two plasmids could support cells’ survival (Fig. 1f). GFP expression increased over time with puromycin selection (Additional file 1: Figure S2B and C). We next tested the capacity of the Tet-Off system for regulation of GFP expression. Three days after addition of Dox, expression of GFP was efficiently shut down (Fig. 1f, Additional file 1: Figure S2B and C).

**Fig. 2:**
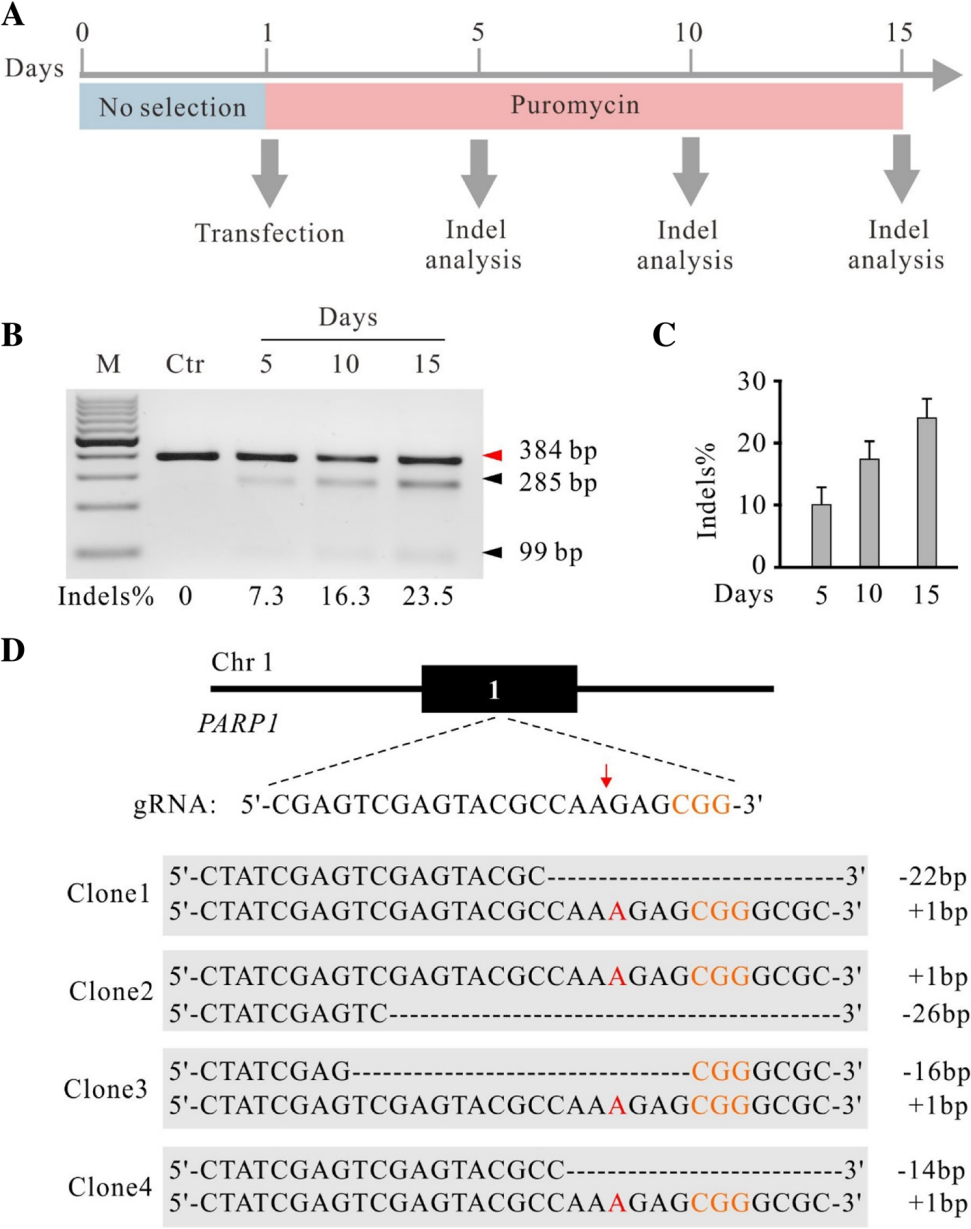
The krCRISPR system enables efficient gene knockout for the *PARP1* gene. **a** Schematic of the experimental workflow. **b** Representative gel pictures of T7E1 assay for detection of indels at *PARP1* sites in HEK293T cells. The indel frequency was labeled below. Ctr is the PCR band from unmodified cells with T7 enzyme digestion. **c** Quantification for the T7E1 assay for Fig. 2b (*n* = 3, error bars showed mean ± SEM). **d** Examples of indel sequences for four single cell-derived clones. Schematic of the gRNA target site was shown above. PAM sequence is marked in orange. Cas9 cutting site is indicated by red arrow. Insertions are indicated by red letter

We next investigated the capacity of the knockout-rescue system for genome editing. We cloned a gRNA targeting exon1 of poly (ADP-ribose) polymerase 1 (*PARP1*) gene into the KO plasmid. The KO plasmid and Rescue plasmid were co-transfected into HEK293T cells with puromycin selection. At day 5, 10 and 15 after transfection, the indel frequency was analyzed by T7E1 assay (Fig. 2a). As expected, the indel frequency increased over time (Fig. 2b and c). We analyzed twenty single cell-derived clones by Sanger sequencing and all of them were biallelic knockout (Fig. 2d, Additional file 1: Figure S3 and Table 1). In summary, we successfully established a double episomal vector system that enabled efficient genome editing. Hereafter, the double episomal vector system was designated krCRISPR (knockout-rescue CRISPR).

**Table 1:** Efficiency of genome editing for single cell-derived clones

	Heterozyote	Homozygous	WT	Total
*PARP1*	5	12	3	20
*DNMT1*	3	16	1	20
*HDAC3*	3	15	2	20

### The krCRISPR enabled knockout of *HDAC3* gene

**Fig. 3:**
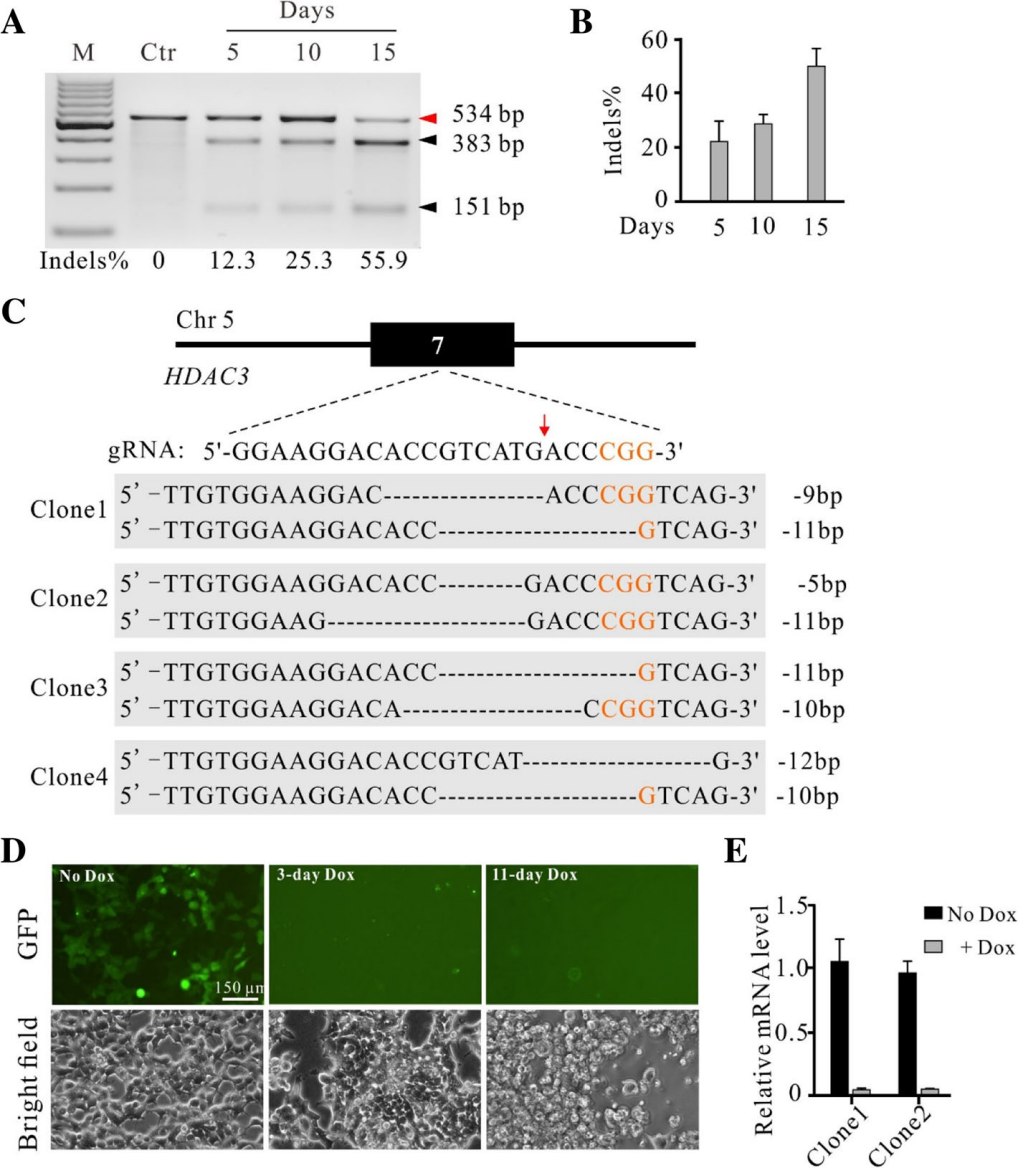
Generation of *HDAC3* knockout-rescue cell lines. **a** Representative gel pictures of T7E1 assay for detection of indels at *HDAC3* sites in HEK293T cells. Ctr is the PCR band from unmodified cells with T7 enzyme digestion. **b** Quantification of the T7E1 assay for Fig. 3a (*n* = 3, error bars show mean ± SEM). **c** Examples of indel sequences for four single cell-derived clones. Schematic of the gRNA target site was shown above. PAM sequence was marked in orange. Cas9 cutting site is indicated by red arrow. **d** Inhibition of exogenous *HDAC3* expression in *HDAC3*-knockout cells caused cell death. The *HDAC3* knockout-rescue cells expressed GFP. Expression of GFP was inhibited by addition of Dox for 3 days. All cells died at day 11. **e** RT-qPCR analysis of the exogenous *HDAC3* expression with or without Dox for two clones (*n* = 3, error bars showed mean ± SEM)

To investigate the capacity of the krCRISPR for essential gene knockout, we used this system to knockout histone deacetylase 3 (*HDAC3*) gene in human HEK293T cells. *HDAC3* is involved in apoptosis, cellular proliferation and DNA damage [19, 20]. Due to the overexpression of *HDAC3* in a variety of cancers, it is an important potential target for cancer [19, 20]. It has been reported that deletion of *HDAC3* is lethal for mouse embryos and mouse embryonic fibroblasts (MEFs) [21, 22]. High-throughput CRISPR/Cas9 screening revealed that deletion of HDAC3 is lethal in several human cell lines [6, 23–25]. A gRNA targeting exon7 of *HDAC3* was cloned into the KO plasmid, and *HDAC3* coding sequence was cloned into the Rescue plasmid. To avoid cleavage by Cas9 nuclease, we created seven point mutations within the gRNA targeting sequence and the Protospacer Adjacent Motif (PAM) sequence that had no effects on the protein sequence (Additional file 1: Figure S4). The KO plasmid and Rescue plasmid were co-transfected into HEK293T cells with puromycin selection. Similar to results for *PARP1*, the indel frequency increased over time (Fig. 3a and b). We analyzed 20 single cell-derived clones by using Sanger sequencing and 15 of them were biallelic knockout (Fig. 3c, Additional file 1: Figure S5 and Table 1). We further investigated the essentiality of the *HDAC3* for cell viability by repressing exogenous *HDAC3* expression in two single cell-derived clones. After 3 days of Dox treatment, *HDAC3* expression was turned off by monitoring GFP expression (Fig. 3d). The results were confirmed by qPCR with primers specifically targeting exogenous *HDAC3* gene (Fig. 3e). Most cells were dead following 11 days of Dox treatment (Fig. 3d). A previous study in mouse embryonic fibroblasts (MEFs) has shown that Hdac3 knockout led to a delay in cell cycle progression, cell-cycle dependent DNA damage, and observed 20–30% of cell death at day 5 after Hdac3 knockout [21]. In summary, these data demonstrated that the krCRISPR technology could knockout genes that are essential for cell survival.

### The krCRISPR enabled knockout of *DNMT1* gene

**Fig. 4:**
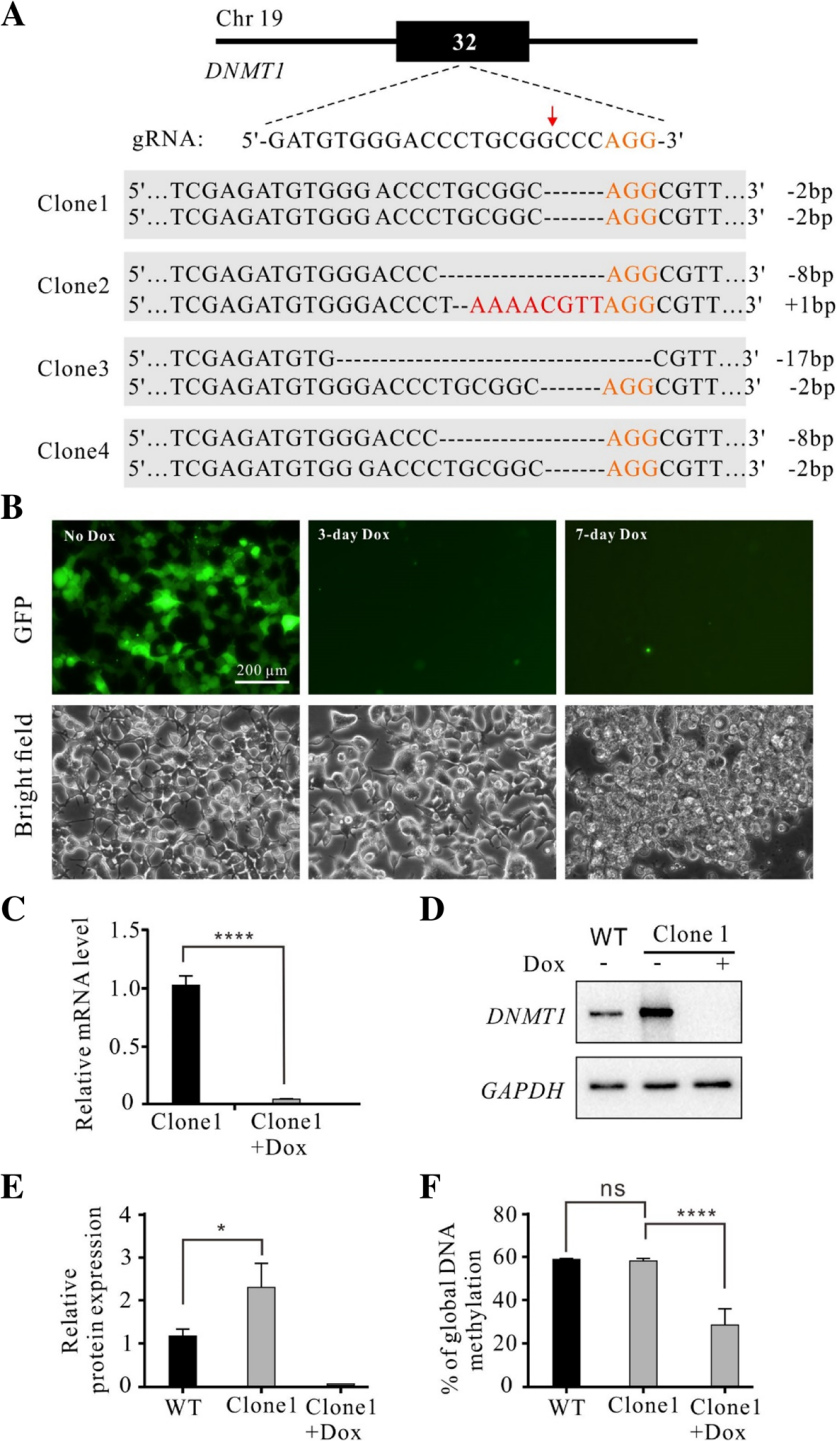
Generation of *DNMT1* knockout-rescue cell lines. **a** Examples of indel sequences for four single cell-derived clones. Schematic of the gRNA target site is shown above. PAM sequence is marked in orange. Cas9 cutting site is indicated by red arrow. Insertions are indicated by red letter. **b** Inhibition of exogenous *DNMT1* expression in *DNMT1*-knockout cells caused cell death. The *DNMT1*knockout-rescue cells expressed GFP. Expression of GFP was inhibited by addition of Dox for 3 days. All cells died at day 7. **c** RT-qPCR analysis of the exogenous *DNMT1* expression with or without Dox for a single cell-derived clone (*n* = 3, error bars showed mean ± SEM). **d** Western blot analysis of total *DNMT1* expression with or without addition of Dox. WT cells were used as a control. **e** Quantification for the Western blot assay for Fig. 4d (*n* = 3, error bars showed mean ± SEM). **f** Luminometric Methylation Assay (LUMA) showed that the global DNA methylation levels were the same for the WT cells and *DNMT1* knockout-rescue cells. Addition of Dox significantly decreased DNA methylation level in *DNMT1* knockout-rescue cells (*n* = 3, error bars showed mean ± SEM)

To demonstrate the capacity of the krCRISPR for essential gene knockout, we showed another example of gene knockout by depleting *DNMT1* which is one of the DNA methyltransferases for maintenance of DNA methylation over replication [26]. Deletion of *DNMT1* is lethal for a variety of dividing somatic cells [3, 27–29]. A gRNA targeting exon32 of *DNMT1* was cloned into the KO plasmid, and *DNMT1* coding sequence was cloned into the Rescue plasmid. To avoid cleavage by Cas9 nuclease, we created five point mutations within the gRNA targeting sequence and the Protospacer Adjacent Motif (PAM) sequence that have no effects on the protein sequence (Additional file 1: Figure S4). The KO and Rescue plasmids were co-transfected into HEK293T cells with puromycin selection for 15 days. Twenty single cell-derived clones were analyzed by Sanger sequencing and 16 of them were biallelic knockout (Fig. 4a, Additional file 1: Figure S6 and Table 1).

We further investigated the effects of *DNMT1* repression on cell survival in two single cell-derived clones. After 3 days of Dox treatment, *DNMT1* expression was shut down by indication of GFP expression (Fig. 4b). At day seven after Dox treatment, the cells started undergoing apoptosis (Fig. 4b). A previous study in human embryonic stem cells has shown that all cells died within 9 days of DNMT1 knockout [3]. RT-qPCR analysis with primers specifically targeting exogenous *DNMT1* gene revealed that *DNMT1* expression was significantly downregulated (Fig. 4c). Western blot showed that the expression of *DNMT1* in clone1 was higher than that in the WT cells at protein level, but it was undetectable after Dox treatment at day seven (Fig. 4d and e). Luminometric methylation assay (LUMA) showed that the methylation level significantly decreased after Dox treatment (Fig. 4f). Altogether, these data demonstrated that we could readily obtain *DNMT1* homozygous mutant cell lines by using the krCRISPR technology.

### The krCRISPR enables to study effects of gene mutations

**Fig. 5:**
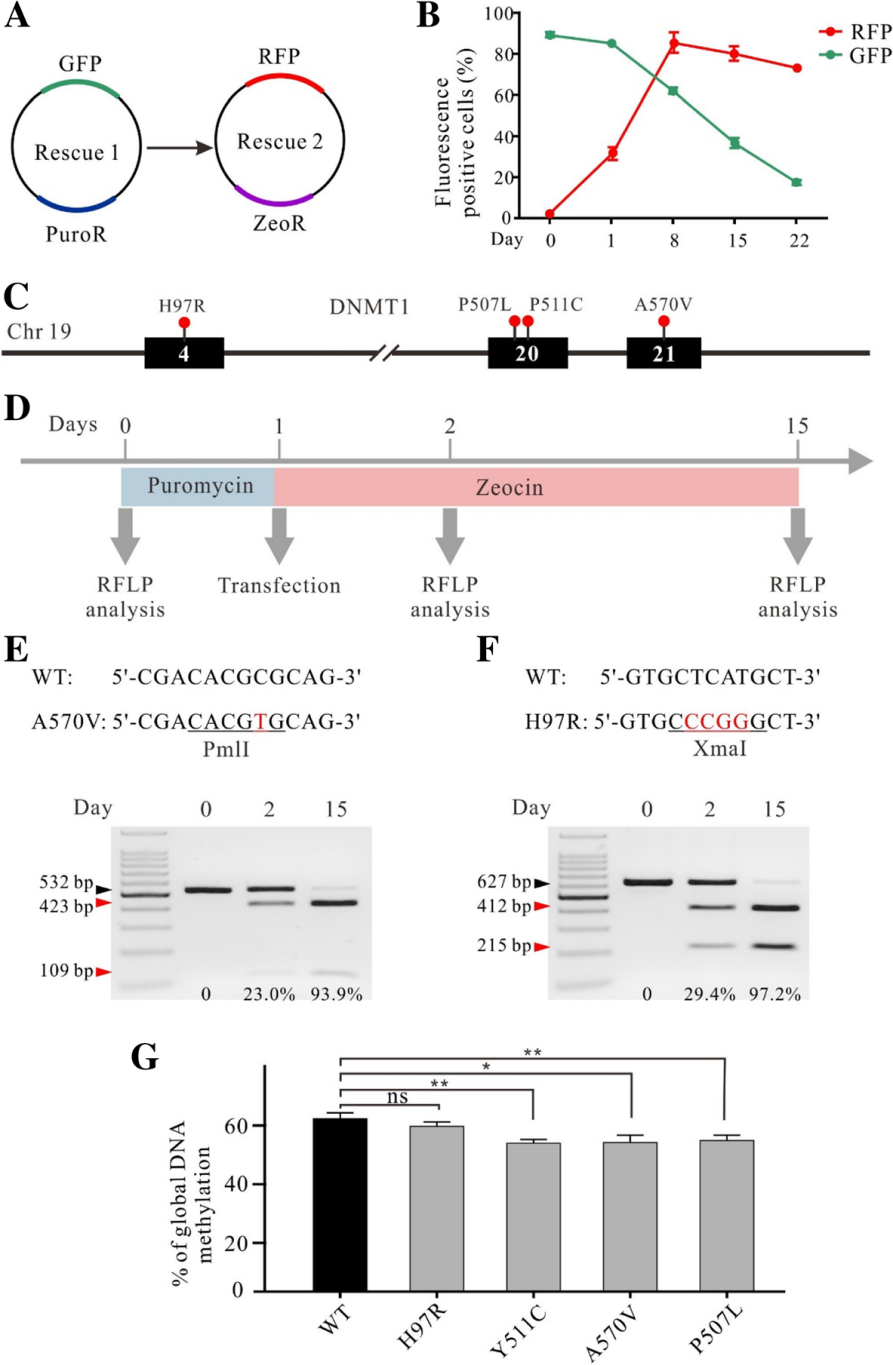
The krCRISPR system enabled analysis of the effects of *DNMT1* mutation on DNA methylation. **a** Schematic of the Rescue1 and Rescue2 plasmid design. Rescue1 plasmid contains a puromycin resistant gene and a GFP gene, while Rescue 2 plasmid contains a zeocin resistant gene and RFP gene. Transfection of Rescue2 plasmid into the knockout-Resuce1 cells will result in replacement of Rescue1 plasmid by Rescue2 plasmid under zeocin selection. **b** Flow cytometry analysis showed that the GFP positive cells were gradually replaced by RFP positive cells over time (*n* = 3, error bars showed mean ± SEM). **c** Distribution of the four *DNMT1* mutations. **d** Schematic of the experimental workflow. **e** and **f** Two examples of Rescue plasmid replacement. The Rescue1 plasmid was gradually replaced by Rescue2 plasmid. A PmlI restriction site for A570V and an XmaI restriction site for H97R were introduced into the Rescue2 plasmids respectively. At day 0, 2 and 15, the plasmid DNA was isolated from cells and PCR-amplified for RFLP analysis. Gene mutations are labeled in red letter; the restriction sites are underlined. Black triangles indicate the Rescue1 plasmid; red triangles indicate the Rescue2 plasmid. **g** The effects of individual mutations on DNA methylation were measured by LUMA. The exogenous *DNMT1* genes encoded by Rescue plasmids were shown below. H97R did not influence DNA methylation level. Y511C, A570V and P507L decreased DNA methylation level (*n* = 3, error bars showed mean ± SEM)

In addition to gene knockout, the krCRISPR also enables us to study the effects of gene mutations. We hypothesized that if we established a knockout-rescue cell line with KO-Rescue1 plasmids, we could use another Rescue plasmid (Rescue2) to replace the original Rescue1 plasmid. Rescue2 could encode genes that contain mutations of interest so that we can study them in the cells. To test whether the Rescue plasmid can be replaced by another plasmid, we designed a Rescue2 plasmid with zeocin resistance gene and RFP marker (Fig. 5a). We transfected this plasmid into cells established in Fig. 1f, where the Rescue plasmid (here we called it Rescue1) encoded GFP and puromycin resistant gene. Under zeocin selection, GFP was gradually replaced by RFP, indicating that the Rescue1 plasmid was replaced by Rescue2 plasmid. At day 22, the percentage of GFP positive cells was only 17.5% and RFP was 72.5% (Fig. 5b and Additional file 1: Figure S7A).

Next, we used this strategy to analyze the effects of four *DNMT1* mutations on DNA methylation (Fig. 5c and Additional file 1: Figure S7B). Among them, H97R is a common variation that is not associated with any known diseases [30–32]. Y511C is associated with hereditary sensory and autonomic neuropathy type 1 with dementia and hearing loss (HSAN1E) [33, 34]; A570V is associated with autosomal dominant cerebellar ataxia, deafness and narcolepsy (ADCA-DN) [35]. Both Y511C and A570V mutations could induce global hypomethylation [33, 34, 36]. P507L is a newly identified mutation associated with HSAN1E [37], but whether it could influence DNA methylation has not been investigated. Individual Rescue plasmids were transfected into the *DNMT1* knockout cells with zeocin selection (Fig. 5d). To facilitate the analysis of plasmid replacement by RFLP, a restriction site was introduced into the *DNMT1* gene without changing the protein sequence of the Rescue2 plasmid (PmlI for A570V; XmaI for H97R) (Fig. 5e and f). For A570V site, the ratio of the Rescue2 to total amount of plasmid DNA was 23.0% at day 2 and 93.9% at day 20, indicating that the Rescue1 plasmid was gradually replaced by Rescue2 (Fig. 5e). For H97R site, the ratio of the Rescue2 to total amount of plasmid DNA was 29.4% at day 2 and 97.2% at day 20 (Fig. 5f).

Next, we performed genome-wide methylation analysis for the individual mutations with LUMA. Compared to control Rescue plasmid expressing WT *DNMT1*, H97R variation did not influence DNA methylation level; Y511C and A570V mutation decreased DNA methylation levels, consistent with previous reports (Fig. 5g) [34, 36]. P507L also decreased DNA methylation level (Fig. 5g), indicating that it could potentially be a disease-causing mutation. In summary, we established a platform that can be used to study the effects of mutations at cellular level.

### Off-target analysis

**Fig. 6:**
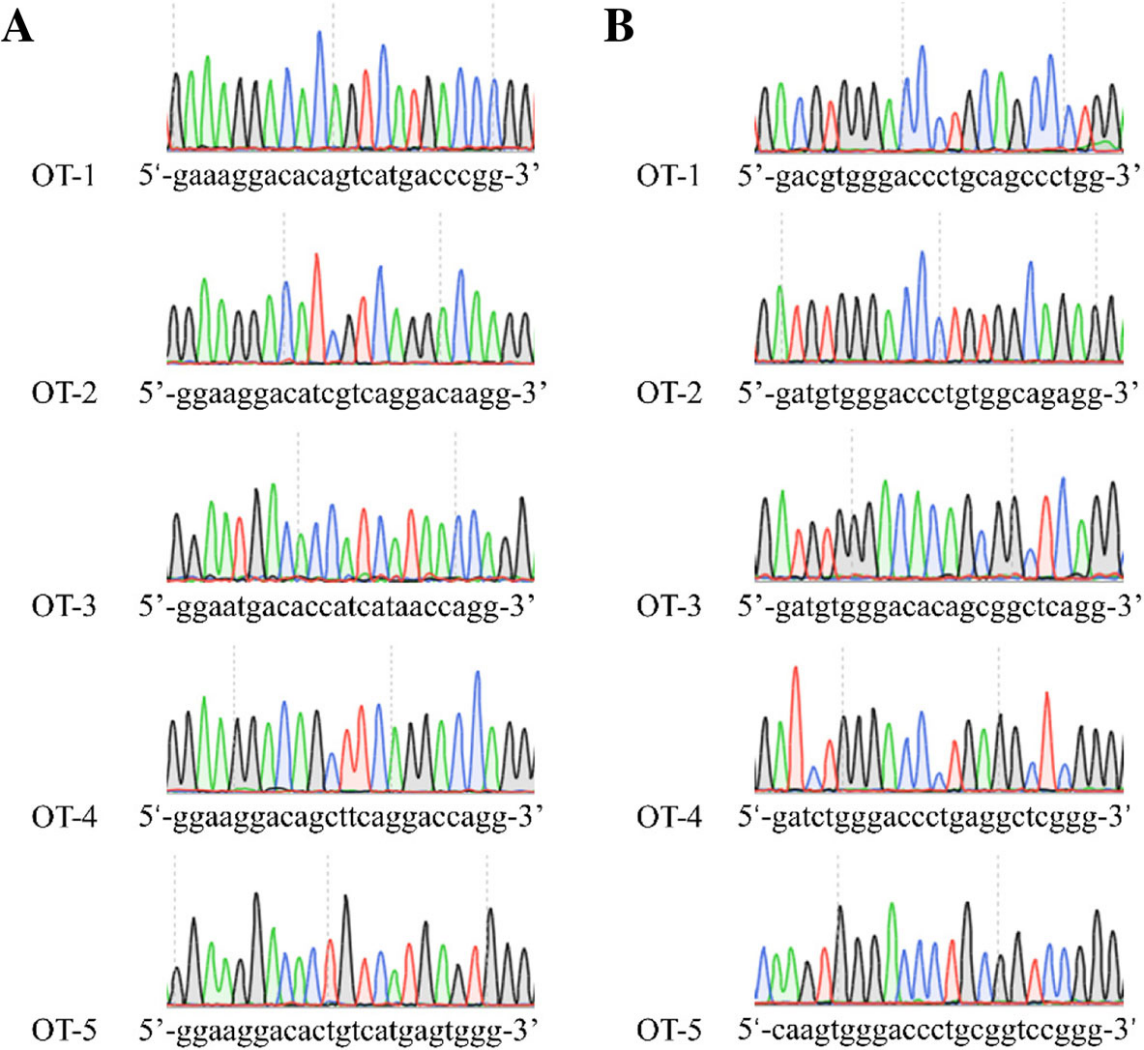
Analysis of potential off-target sites. **a** Five potential off-target sites for clone1 of *HDAC3* were sequenced. **b** Five potential off-target sites for clone1 of *DNMT1* were sequenced

Off-target mutations are often generated during genome editing [38, 39]. The krCRISPR system requires long-term editing which may increase off-target effects. We used an online tool (http://www.rgenome.net/cas-offinder/) to search for potential off-target sites and selected five top ranked potential off-target sites for gRNA-HDAC3. These potential off-targets have two or three mismatches compared to the targeting sequence (Table 2). We tested these sites in two *HDAC3* knockout-rescue clones that were derived from a single cell, but we did not observe off-target mutations (Fig. 6a and Additional file 1: Figure S8A). We also analyzed five potential off-target sites for *DNMT1* knockout-rescue clones, but we did not observe off-target mutations (Fig. 6b, Additional file 1: Figure S8B and Table 3). Notably, we could not exclude that off-target mutations occurred somewhere in the genome. Whole-genome sequencing will be desirable for further detection of off-target mutations in the future.

**Table 2:**
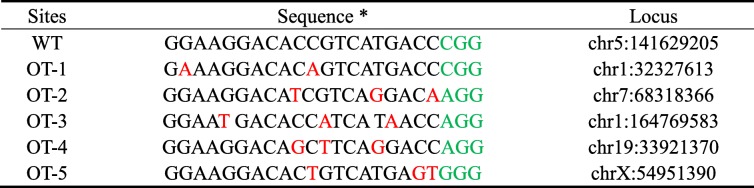
Potential off-target sequences for *HDAC3*

*PAM sequences were marked in green and the mismatched nucleotides were labeled in red

**Table 3:**
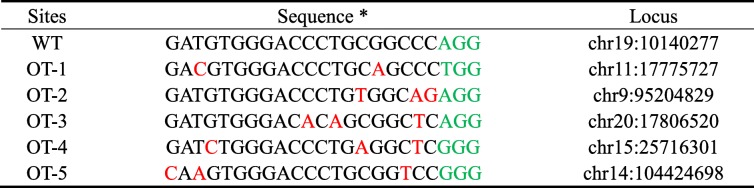
Potential off-target sequences for *DNMT1*

*PAM sequences were marked in green and the mismatched nucleotides were labeled in red

## Discussion

In this study, we demonstrated a simple and efficient method to knockout essential genes in cell lines using krCRISPR technology. This technology only requires two steps to obtain knockout-rescue cell lines: i) clone the gRNA into the KO vector and rescuing gene into the Rescue vector; ii) co-transfect both plasmids into cells and select single cell-derived clones with biallelic frame shift mutations. The expression of rescue genes can be efficiently turned off by Tet-Off technology, allowing the effects of gene knockout on cells to be studied. The krCRISPR enables efficient knockout of endogenous genes due to the puromycin selection and long-term genome editing [17]. In contrast, previous strategies for essential gene knockout are time-consuming and labor-extensive, requiring multiple steps to generate stable integration cell lines that contain either Tet-Off elements or Cre/loxP elements [3, 7–9, 40, 41].

In addition to gene knockout, the krCRISPR also enables users to study the effects of mutations on cells. Each human is estimated to carry on average ~ 60 de novo point mutations that arose in the germ line of their parents [42]. These mutations are the principal cause of heritable disease. Furthermore, genome-wide association studies (GWAS) have identified a large number of somatic mutations that are associated with cancers [43, 44]. Although CRISPR/Cas9 technology allows efficient introduction of specific mutations into the endogenous loci [45, 46], it is time-consuming. The krCRISPR offers an alternative strategy to study gene mutations in cells. One can knockout endogenous genes and express rescue genes containing mutation of interest simultaneously. Once the knockout-rescue cell lines are established, the rescue plasmid can be replaced by other rescue plasmid containing different mutations. In summary, the krCRISPR technology offers a platform that facilitates the study of essential genes’ functions.

## Conclusions

In conclusion, we developed a double episomal vector system that allows generation of inducible knockout-rescue cell lines. In this system, one vector expresses gRNA and Cas9 nuclease, and the other vector expresses an inducible rescue gene. Users can easily knockout an essential gene by the expression of corresponding gRNA and rescue gene.

## Materials and methods

### Cell culture and transfection

The HEK293T cell line (ATCC) was grown in Dulbecco’s Modified Eagle Medium (DMEM), supplemented with 10% fetal bovine serum (Gibco), 1x penicillin-streptomycin and passaged using 0.25% Trypsin-EDTA every other day. Cells were incubated at 37 °C with 5% CO_2_.

### Plasmids

Knockout (KO) plasmid: this plasmid was modified from epiCRISPR plasmid [17]. The SpCas9 and tTA were co-expressed from an EF1α promoter as a single protein separated by self-cleaving P2A peptides; gRNA was expressed from a human U6 promoter. The synthetic oligonucleotide duplexes encoding gRNAs can be cloned into BspQI restriction sites. The sequence of the plasmid is available in Additional file 1: Figure S9.

The Rescue plasmid: copGFP and puromycin resistance genes were co-expressed from a pTRE promoter separated by two P2A peptides. The exogenous gene can be cloned into multiple cloning sites downstream of pTRE promoter. The sequence of the plasmid is available in Additional file 1: Figure S10. cDNA of DNMT1, PARP1 and HDAC3 was synthesized by GENWIZ (China) and inserted into KpnI/ AsisI restriction sites of the Rescue plasmid. Notably, synonymous mutations were introduced on gRNA targeting sequence to prevent Cas9 cleavage.

The Rescue2 plasmid: this plasmid is similar to Rescue plasmid except that copGFP-puro cassette was replaced with RFP-zeo cassette. The sequence of the plasmid is available in Additional file 1: Figure S11. The *DNMT1*^*P*507L^*DNMT1*^*H*97R^, *DNMT1*^*A*570v^ and *DNMT1*^*Y5*11C^ mutations were created on the *DNMT1*^*WT*^ using Gibson Assembly Cloning Kit (#E5510S, NEB) according to the online protocol. The primers and oligonucleotides used in this study are shown in Additional file 1: Table S1.

### Transfection, T7 assay and sequencing analysis for genome modification

HEK293T cells were seeded on 12-well plates in 500uL of growth medium without antibiotics. After 24 h, HEK293T cells were transfected at 60–70% confluency using Lipofectamine 2000 transfection regent (Invitrogen) according to the manufacturer’s protocol. For double-plasmid transfection, 500 ng of KO plasmid and 500 ng of Rescue plasmid were transfected per well. From day 2, cells were selected by puromycin (1.5–2 μg/mL). At day 5, 10 and 15, genomic DNA was extracted from cells using QuickExtract (Cat. # QE09050, Lucigen) following the manufacturer’s instructions and T7E1 assay was performed according to a previously described method [10]. Briefly, genomic region containing the gRNA target site was PCR-amplified using Q5 High-Fidelity DNA polymerase (NEB) and the PCR products were purified using QIAquick Gel Extraction Kit (28,706, QIAGEN). A total of 300 ng purified PCR products were re-annealed and digested with T7E1 enzyme (#M0302S, NEB) for 30 min at 37 °C. The PCR products were analyzed on 1.5% agarose gels. Gels were imaged with EB staining and quantified using ImageJ software according to the band intensities. To analyze indel sequences for single cell-derived clones, cells were digested to single cells 10 days after transfection and seeded into 96-well plates using flow cytometer. A week later, genomic DNA of clones was extracted for PCR-amplification. The PCR products containing gRNA targeting sites were cloned into T-vector (A1410, Promega) according to the manufacturer’s instructions for Sanger sequencing analysis. For plasmid replacement assay, 1μg of Rescue2 plasmid was transfected into knockout-rescue cells and selected with zeocin (300-400μg/mL) from day 2.

### Protein extraction and Western blotting

Cell samples were harvested and lysed with NP-40 buffer (Beyotime) in the presence of 1 mM Phenylmethanesulfonyl fluoride (Beyotime). After centrifugation at 12000 rpm for 10 min in a 4 °C pre-cooled centrifuge, the supernatant was collected for Western blot analysis. Proteins were separated by 8% SDS-PAGE gel and then transferred to a polyyinylidene fluoride (PVDF) membrane (Thermo). After blocking with 5% (wt/vol) BSA (Sigma) in TBS-T (0.1% Tween 20 in 1x TBS) buffer for 1 h at room temperature, the membrane was incubated with primary antibodies at 4 °C overnight. Antibodies used include: anti-DNMT1 (1:1000; ab13537 Abcam) and anti-GAPDH (1:2000, 5174S, Cell Signaling). After three washes with TBS-T of 5 min each, the membranes were incubated with secondary antibody (1:10,000; ab6721 Abcam) at room temperature for 1 h, followed by three washes and imaged.

### RNA isolation and quantitative reverse transcription polymerase chain reaction

Total RNA was extracted from cells using Trizol regent (Ambion) following the manufacturer’s instructions. First-strand cDNA was synthesized from the isolated RNA using 5x All-In-One RT MasterMix kit (Cat. No. G492, abm) according to the manufacturer’s manual. 2x SYBR Green qPCR Master Mix (Cat. No. 21703, bimake) was used to quantify the expression of HDAC3 and DNMT1 mRNA. GAPDH was used as an internal control for normalization. The primers were designed for amplification of exogenous gene in plasmid using Primer Premier 5.0. All primer sequences used are shown in Additional file 1: Table S1. The qRT-PCR was performed using Bio-Rad Real-Time PCR. Detection System and the relative expression level was calculated using the 2-ΔΔCt method.

### Flow cytometry

Cells for flow cytometry analysis were treated with 0.25% Trypsin-EDTA, washed twice and resuspended in 300uL PBS. The percentage of GFP and RFP positive cells was quantified using flow cytometer (Gallios, Beckman Coulter) according to the manufacturer’s protocol. Data were analyzed using FlowJo software.

### Luminometric methylation assay (LUMA)

Firstly, total DNA was purified with phenol: chloroform. Briefly, cells were harvested and washed twice with PBS. Cells were then resuspended in 460uL of nuclear lysis buffer, 20uL of proteinase K (20 mg/mL) and 20uL of 10% SDS followed by incubation at 58 °C overnight. 5uL RNaseA (10 mg/mL) was then added, mixed by vortexing and incubated for 3 h at 37 °C. 500uL phenol:chloroform was added, mixed by vortexing and incubated for 3 min at room temperature following centrifuged for 20 min at 13000 rpm. 400uL of aqueous phase containing DNA was then transferred to a fresh tube followed by the addition of 400uL isopropanol and vortexed for 30s, and finally 40uL NaAc (pH = 5.2) was added and vortexed for 2 min. The remaining DNA precipitate was washed twice with 75% ethanol, and dissolved in 80uL ddH_2_O.

Subsequently, pyrosequencing was performed. 400 ng genomic DNA was cleaved with HpaII + EcoRI or MspI + EcoRI in two separate 20uL reactions containing 400 ng DNA, 1uL HpaII (or 0.5uL MspI), 0.5uL EcoRI-HF and 2uL 10x cutsmat buffer (NEB). The reactions were incubated at 37 °C for 4 h. Then pyrosequencing was performed following a previously described protocol [47]. The percentage of methylation was calculated based on the LUMA results with the following formula:

Methylation % = 100[1-(HpaII/EcoRI/MspI/EcoRI)].

### Statistical analysis

In this study, statistical analysis was performed using GraphPad Prism 5. All data were presented as mean ± SEM. The unpaired Student’s t-test was adopted to determine the statistical differences between the samples of two groups. Significant levels: *P < 0.05, **P < 0.01, ****P < 0.001. All experiments were repeated three times independently.

## Additional file

Additional file 1: **Figure S1.** Strategies for knockout of essential genes in cells. **Figure S2.** Establishment of the knockout-rescue system with double episomal vectors. **Figure S3.** Indel sequences at *PARP1* site. **Figure S4.** The gRNA targeting sequences on chromosome and their corresponding sequences on the rescue genes. **Figure S5.** Indel sequences at *HDAC3* site. **Figure S6.** Indel sequences at *DNMT1* site. **Figure S7.** Results of flow cytometry. **Figure S8.** Analysis of potential off-target sites. **Figure S9** KO plasmid sequence. **Figure S10.** Rsecue plasmid sequence. **Figure S11** Rsecue2 plasmid sequence. **Table S1.** Primers and oligonucleotides. (DOCX 3386 kb)

## Abbreviations

DSBs: Double strand breaks
EBNA1: Epstein-Barr-associated nuclear antigen 1
EBV: Epstein-Barr virus
gRNA: guide RNA
HDR: Homology-directed repair
krCRISPR: knockout-rescue CRISPR
NHEJ: Non-homologous end-joining
PAM: Protospacer Adjacent Motif
RFLP: Restriction fragment length polymorphism
ssDNA: single-stranded DNA
