# Supplementary material for: krCRISPR: an easy and efficient strategy for generating conditional knockout of essential genes in cells
Source: J Biol Eng. 2019 Apr 24;13:35. doi: 10.1186/s13036-019-0150-y (PMC6480908; doi:10.1186/s13036-019-0150-y)
Supplement: Supplementary file 1 — Figure S1. Strategies for knockout of essential genes in cells. Figure S2. Establishment of the knockout-rescue system with double episomal vectors. Figure S3. Indel sequences at PARP1 site. Figure S4. The gRNA targeting sequences on chromosome and their corresponding sequences on the rescue genes. Figure S5. Indel sequences at HDAC3 site. Figure S6. Indel sequences at DNMT1 site. Figure S7. Results of flow cytometry. Figure S8. Analysis of potential off-target sites. Figure S9 KO plasmid sequence. Figure S10. Rsecue plasmid sequence. Figure S11 Rsecue2 plasmid sequence. Table S1. Primers and oligonucleotides. (DOCX 3386 kb) [file 13036_2019_150_MOESM1_ESM.docx]

**
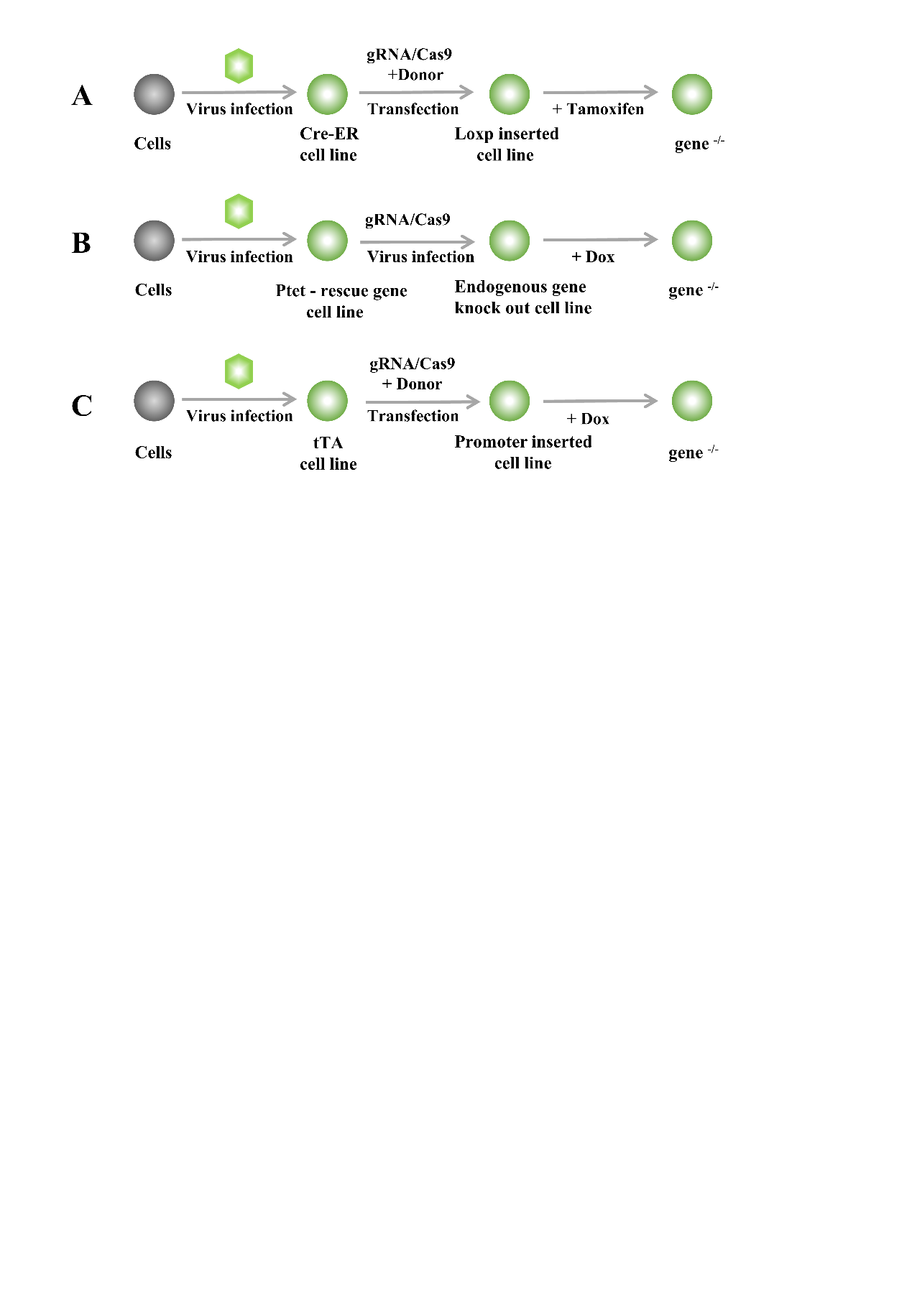
**

**Fig. S1. Strategies for knockout of essential genes in cells.** (A) Cre/loxP recombination system. Firstly, a transgenic cell line expressing Cre-ER is generated. Then, a pair of 34 bp loxP sites flanking the target genes is inserted through CRISPR-based homologous recombination. The Cre recombinase enters nucleus after tamoxifen treatment, causing gene deletion between the two loxP sites. (B) Firstly, a transgenic cell line expressing a rescue gene under the control of Ptet promoter (Doxycycline-inducible gene expression) is generated through lentivirus infection. Then, the endogenous target gene is knocked out. The expression of the rescue gene can be shut down by doxycycline (Dox) treatment. (C) Firstly, a transgenic cell line expressing tetracycline transactivator (tTA) is generated and a TRE-CMV promoter is inserted to upstream of the endogenous target gene. The expression of the endogenous gene can be shut down by Dox treatment.


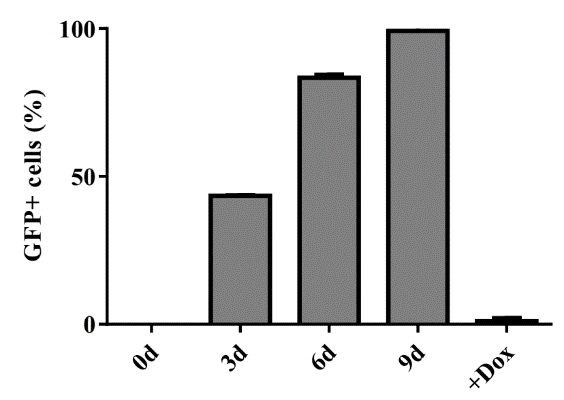
**A C**


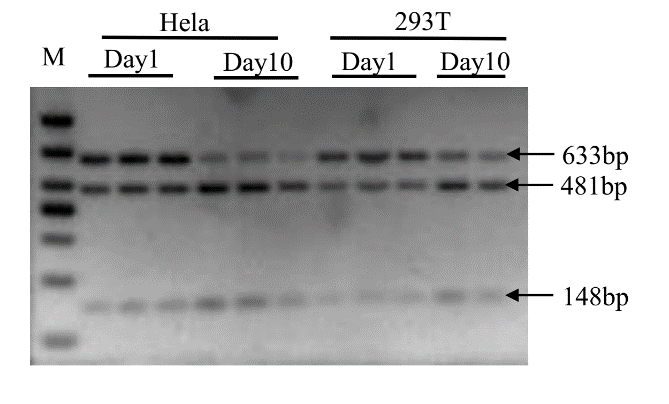


**B**


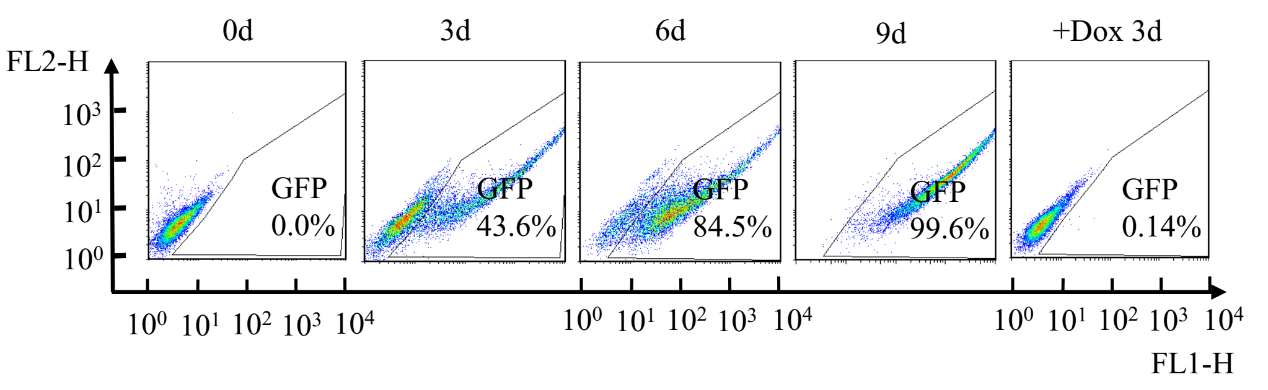


**Fig. S2. Establishment of the knockout-rescue system with double episomal vectors.** (A) Representative gel pictures of RFLP analysis of double plasmids at day 1 and day 10 after transfection in Hela and HEK293T cells. An MfeI restriction site is only present on the KO plasmid and digestion of the PCR products resulted in two bands (481+148 bp). A 633 bp fragment amplified from Rescue plasmid could not be digested by MfeI. (B) Results of flow cytometry. Cells transfected with double plasmids were analyzed by flow cytometry at day 3, 6 and 9. Cells treated with Dox for three days reduced GFP expression. X-axis : FL1-H; Y-axis : FL2-H. (C) Quantification of GFP-positive cells for S1B using FlowJo software (n=3).


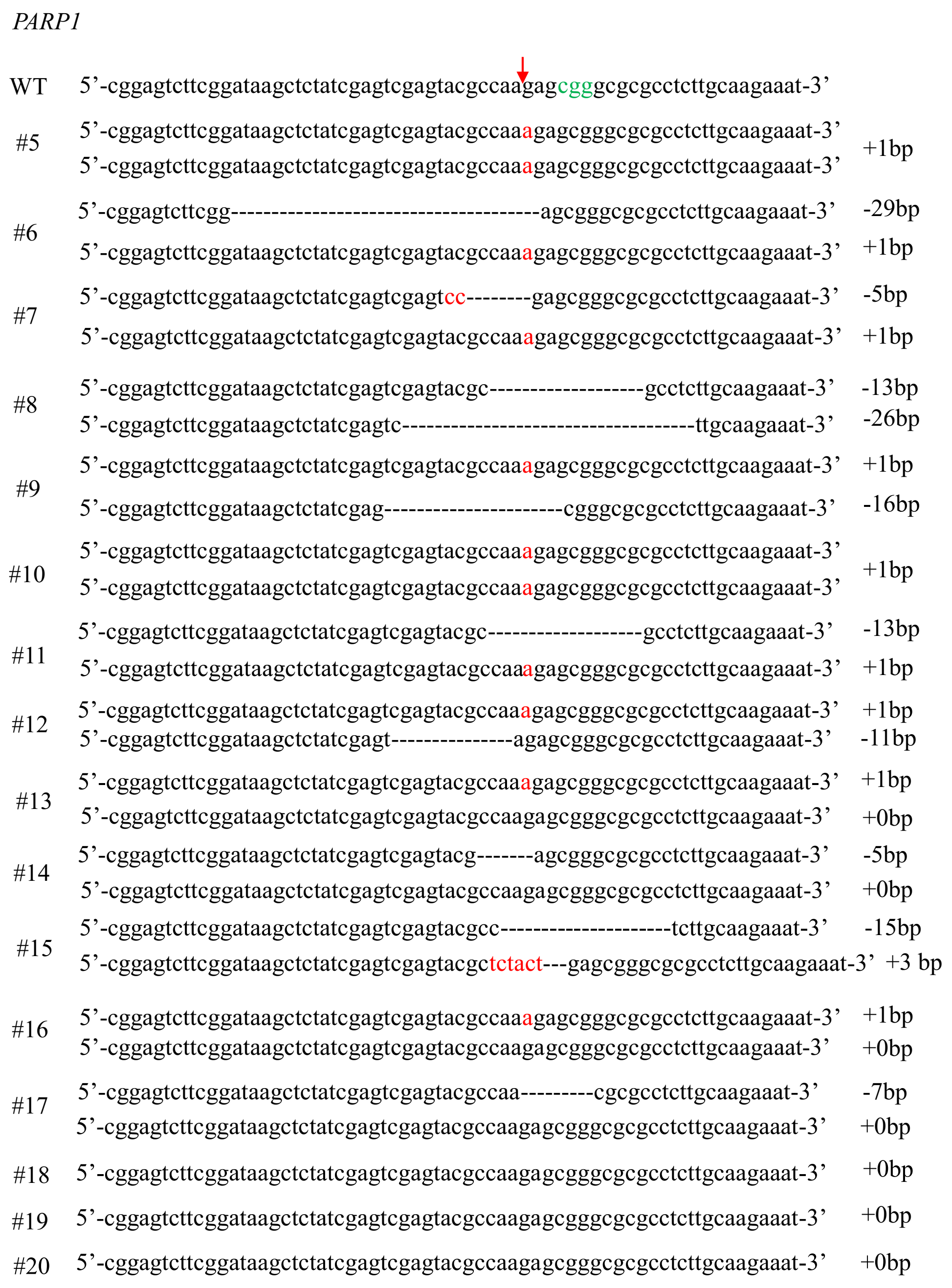


**Fig. S3. Indel sequences at *PARP1* site.** Indel sequences of sixteen single cell-derived clones were shown here. PAM sequence was marked in green. Cas9 cutting site was indicated by a red arrow. Insertions were labeled in red.


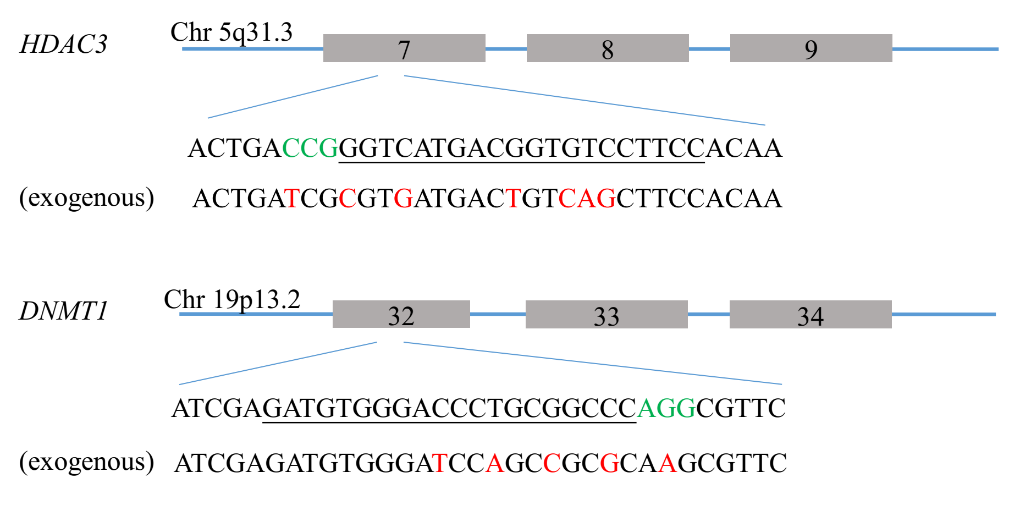


**Fig. S4.** **The gRNA targeting sequences on chromosome and their corresponding sequences on the rescue genes.** The mutations were introduced into the rescue genes so that they cannot be digested by CRISPR/Cas9. The sgRNA targeting sequence is underlined; the PAM sequences were marked in green and mutations were labeled in red.


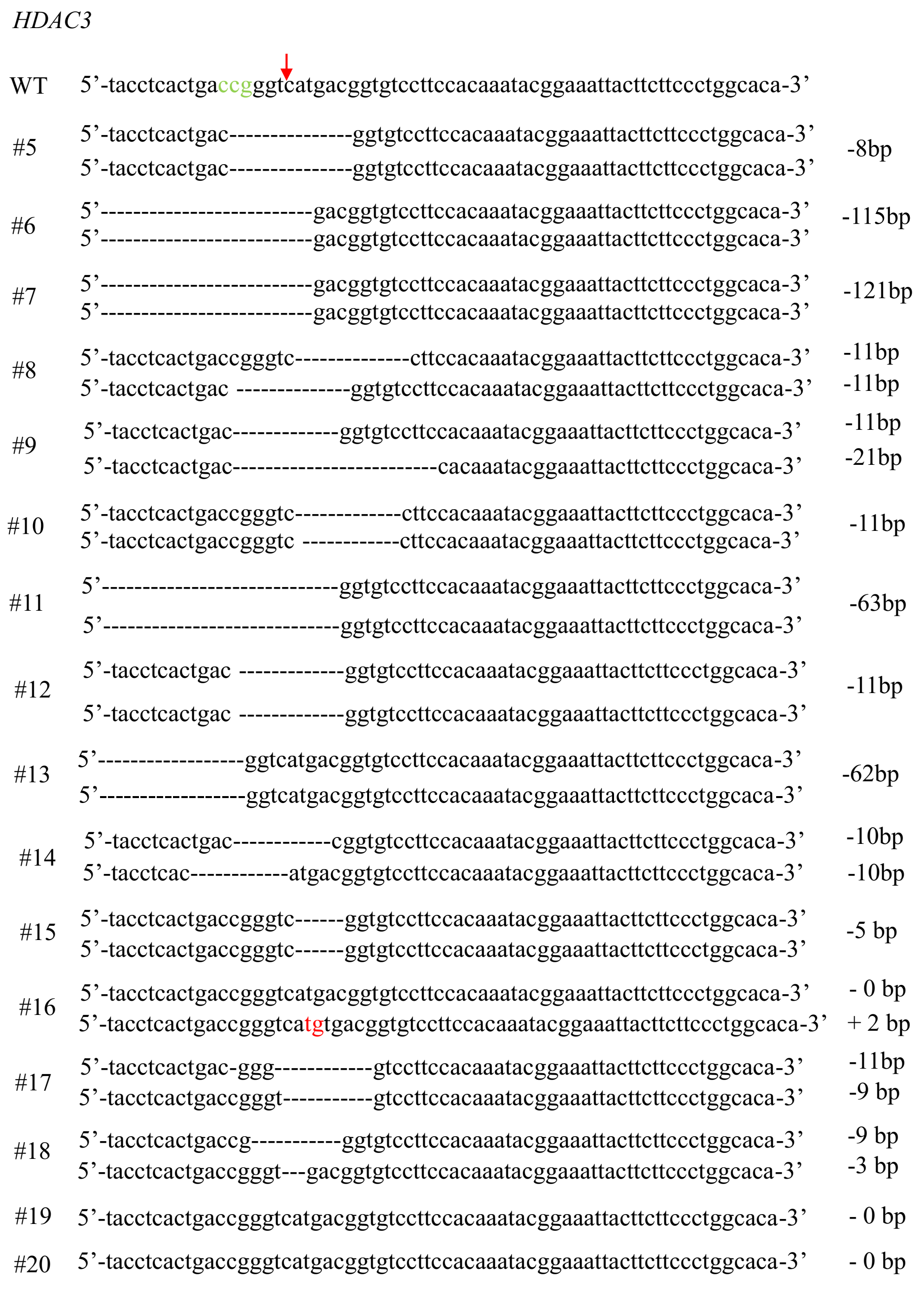


**Fig. S5. Indel sequences at *HDAC3* site.** Indel sequences of sixteen single cell-derived clones were shown here. PAM sequence was marked in green. Cas9 cutting site was indicated by a red arrow. Insertions were labeled in red.


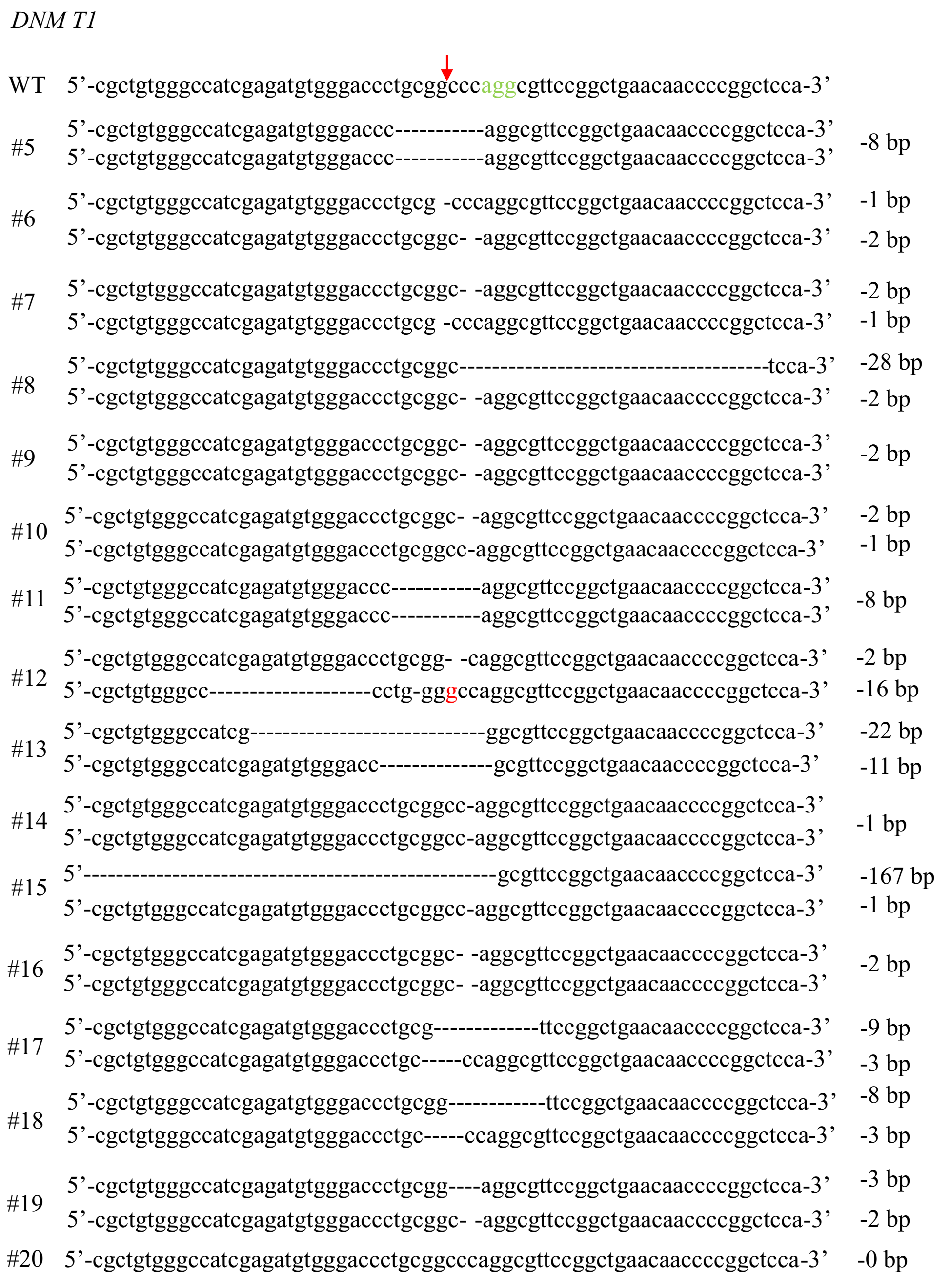


**Fig. S6.** **Indel sequences at *DNMT1* site.** Indel sequences of sixteen single cell-derived clones were shown here. PAM sequence was marked in green. Cas9 cutting site was indicated by a red arrow. Insertions were labeled in red.

**A**


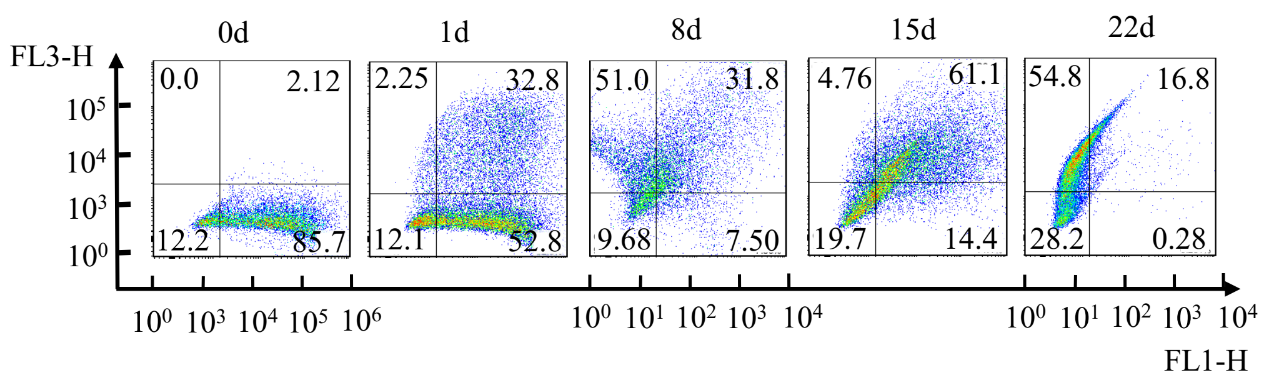


**B**


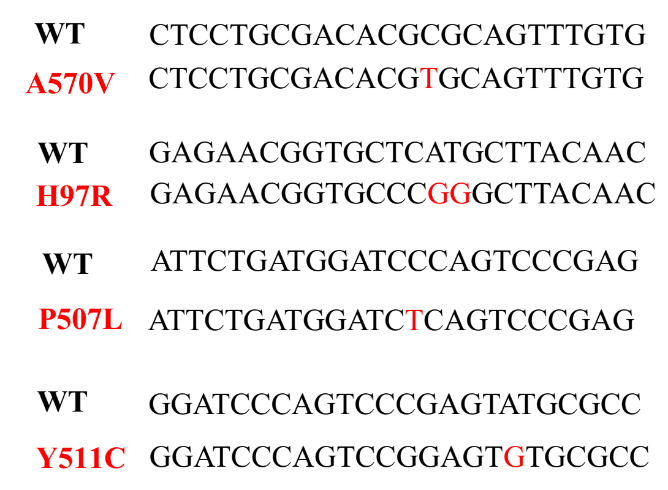


**Fig. S7. Results of flow cytometry.** (A) GFP-positive or RFP-positive cells were analyzed by flow cytoemtry at day 0, 1, 8, 15 and 22 respectively. (B) The sequences of the mutations on the *DNMT1* gene. Mutations were labeled in red.

**
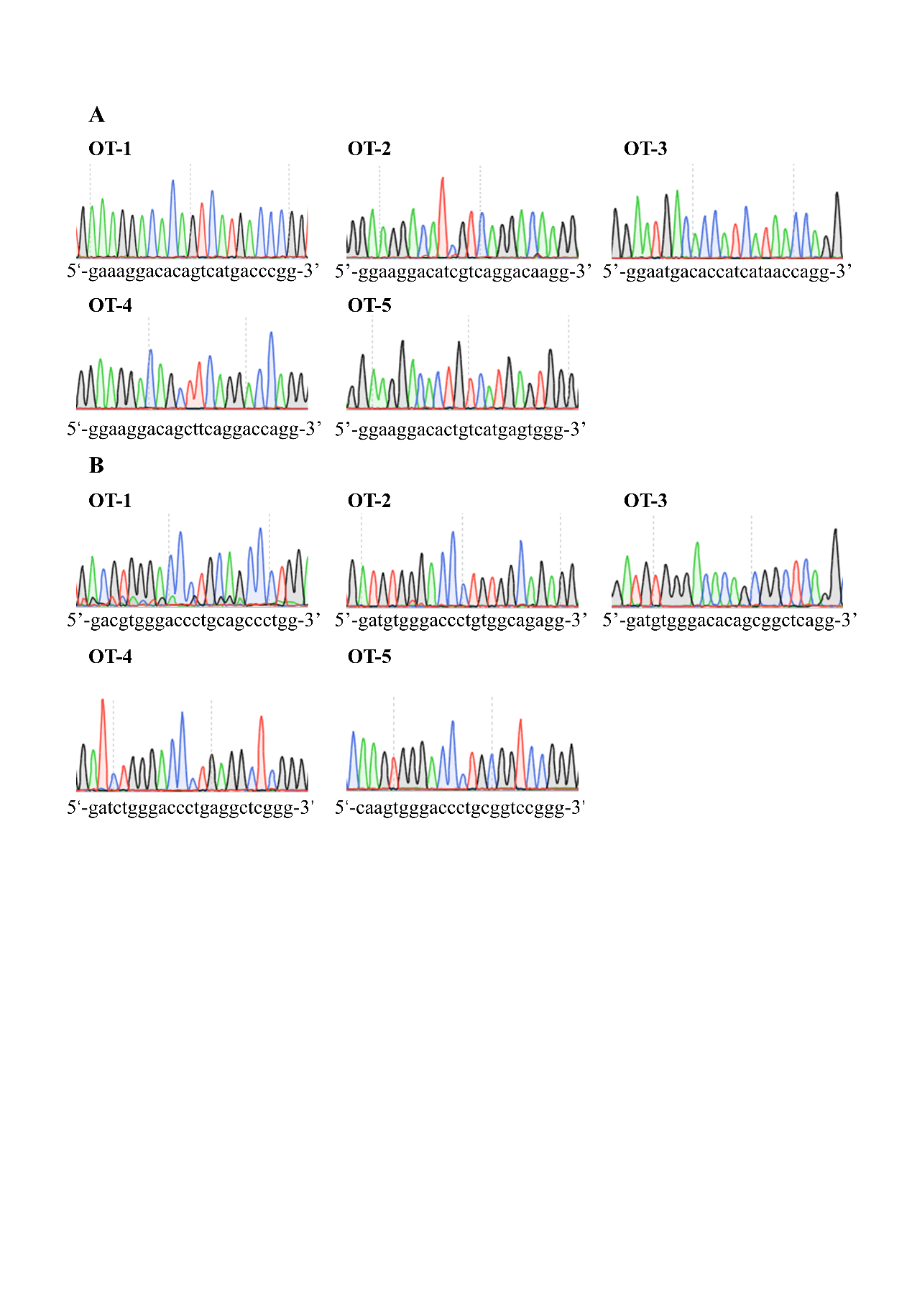
**

**Fig. S8. Analysis of potential off-target sites.** (A) Five potential off-target sites for clone2 of *HDAC3* were sequenced. (B) Five potential off-target sites for clone2 of *DNMT1* were sequenced.

cattatggtacctttcagacccacctcccaaccccgaggggacccagagagggcctatttcccatgattccttcatatttgcatatacgatacaaggctgttagagagataattagaattaatttgactgtaaacacaaagatattagtacaaaatacgtgacgtagaaagtaataatttcttgggtagtttgcagttttaaaattatgttttaaaatggactatcatatgcttaccgtaacttgaaagtatttcgatttcttggctttatatatcttgtggaaaggacgaaacaccgagaagagcgatgctcttcggttttagagctagaaatagcaagttaaaataaggctagtccgttatcaacttgaaaaagtggcaccgagtcggtgcttttttgaattctagatcttgagacaaatggcagtattcatccacaattttaaaagaaaaggggggattggggggtacagtgcaggggaaagaatagtagacataatagcaacagacatacaaactaaagaattacaaaaacaaattacaaaaattcaaaattttcgggtttattacagggacagcagagatccactttggcgccggctcgagtggctccggtgcccgtcagtgggcagagcgcacatcgcccacagtccccgagaagttggggggaggggtcggcaattgaaccggtgcctagagaaggtggcgcggggtaaactgggaaagtgatgtcgtgtactggctccgcctttttcccgagggtgggggagaaccgtatataagtgcagtagtcgccgtgaacgttctttttcgcaacgggtttgccgccagaacacaggtgtcgtgacgcgggatccgccaccatggattacaaagacgatgacgataagatggccccaaagaagaagcggaaggtcggtatccacggagtcccagcagccgacaagaagtacagcatcggcctggacatcggcaccaactctgtgggctgggccgtgatcaccgacgagtacaaggtgcccagcaagaaattcaaggtgctgggcaacaccgaccggcacagcatcaagaagaacctgatcggagccctgctgttcgacagcggcgaaacagccgaggccacccggctgaagagaaccgccagaagaagatacaccagacggaagaaccggatctgctatctgcaagagatcttcagcaacgagatggccaaggtggacgacagcttcttccacagactggaagagtccttcctggtggaagaggataagaagcacgagcggcaccccatcttcggcaacatcgtggacgaggtggcctaccacgagaagtaccccaccatctaccacctgagaaagaaactggtggacagcaccgacaaggccgacctgcggctgatctatctggccctggcccacatgatcaagttccggggccacttcctgatcgagggcgacctgaaccccgacaacagcgacgtggacaagctgttcatccagctggtgcagacctacaaccagctgttcgaggaaaaccccatcaacgccagcggcgtggacgccaaggccatcctgtctgccagactgagcaagagcagacggctggaaaatctgatcgcccagctgcccggcgagaagaagaatggcctgttcggcaacctgattgccctgagcctgggcctgacccccaacttcaagagcaacttcgacctggccgaggatgccaaactgcagctgagcaaggacacctacgacgacgacctggacaacctgctggcccagatcggcgaccagtacgccgacctgtttctggccgccaagaacctgtccgacgccatcctgctgagcgacatcctgagagtgaacaccgagatcaccaaggcccccctgagcgcctctatgatcaagagatacgacgagcaccaccaggacctgaccctgctgaaagctctcgtgcggcagcagctgcctgagaagtacaaagagattttcttcgaccagagcaagaacggctacgccggctacattgacggcggagccagccaggaagagttctacaagttcatcaagcccatcctggaaaagatggacggcaccgaggaactgctcgtgaagctgaacagagaggacctgctgcggaagcagcggaccttcgacaacggcagcatcccccaccagatccacctgggagagctgcacgccattctgcggcggcaggaagatttttacccattcctgaaggacaaccgggaaaagatcgagaagatcctgaccttccgcatcccctactacgtgggccctctggccaggggaaacagcagattcgcctggatgaccagaaagagcgaggaaaccatcaccccctggaacttcgaggaagtggtggacaagggcgcttccgcccagagcttcatcgagcggatgaccaacttcgataagaacctgcccaacgagaaggtgctgcccaagcacagcctgctgtacgagtacttcaccgtgtataacgagctgaccaaagtgaaatacgtgaccgagggaatgagaaagcccgccttcctgagcggcgagcagaaaaaggccatcgtggacctgctgttcaagaccaaccggaaagtgaccgtgaagcagctgaaagaggactacttcaagaaaatcgagtgcttcgactccgtggaaatctccggcgtggaagatcggttcaacgcctccctgggcacataccacgatctgctgaaaattatcaaggacaaggacttcctggacaatgaggaaaacgaggacattctggaagatatcgtgctgaccctgacactgtttgaggacagagagatgatcgaggaacggctgaaaacctatgcccacctgttcgacgacaaagtgatgaagcagctgaagcggcggagatacaccggctggggcaggctgagccggaagctgatcaacggcatccgggacaagcagtccggcaagacaatcctggatttcctgaagtccgacggcttcgccaacagaaacttcatgcagctgatccacgacgacagcctgacctttaaagaggacatccagaaagcccaggtgtccggccagggcgatagcctgcacgagcacattgccaatctggccggcagccccgccattaagaagggcatcctgcagacagtgaaggtggtggacgagctcgtgaaagtgatgggccggcacaagcccgagaacatcgtgatcgaaatggccagagagaaccagaccacccagaagggacagaagaacagccgcgagagaatgaagcggatcgaagagggcatcaaagagctgggcagccagatcctgaaagaacaccccgtggaaaacacccagctgcagaacgagaagctgtacctgtactacctgcagaatgggcgggatatgtacgtggaccaggaactggacatcaaccggctgtccgactacgatgtggaccatatcgtgcctcagagctttctgaaggacgactccatcgacaacaaggtgctgaccagaagcgacaagaaccggggcaagagcgacaacgtgccctccgaagaggtcgtgaagaagatgaagaactactggcggcagctgctgaacgccaagctgattacccagagaaagttcgacaatctgaccaaggccgagagaggcggcctgagcgaactggataaggccggcttcatcaagagacagctggtggaaacccggcagatcacaaagcacgtggcacagatcctggactcccggatgaacactaagtacgacgagaatgacaagctgatccgggaagtgaaagtgatcaccctgaagtccaagctggtgtccgatttccggaaggatttccagttttacaaagtgcgcgagatcaacaactaccaccacgcccacgacgcctacctgaacgccgtcgtgggaaccgccctgatcaaaaagtaccctaagctggaaagcgagttcgtgtacggcgactacaaggtgtacgacgtgcggaagatgatcgccaagagcgagcaggaaatcggcaaggctaccgccaagtacttcttctacagcaacatcatgaactttttcaagaccgagattaccctggccaacggcgagatccggaagcggcctctgatcgagacaaacggcgaaaccggggagatcgtgtgggataagggccgggattttgccaccgtgcggaaagtgctgagcatgccccaagtgaatatcgtgaaaaagaccgaggtgcagacaggcggcttcagcaaagagtctatcctgcccaagaggaacagcgataagctgatcgccagaaagaaggactgggaccctaagaagtacggcggcttcgacagccccaccgtggcctattctgtgctggtggtggccaaagtggaaaagggcaagtccaagaaactgaagagtgtgaaagagctgctggggatcaccatcatggaaagaagcagcttcgagaagaatcccatcgactttctggaagccaagggctacaaagaagtgaaaaaggacctgatcatcaagctgcctaagtactccctgttcgagctggaaaacggccggaagagaatgctggcctctgccggcgaactgcagaagggaaacgaactggccctgccctccaaatatgtgaacttcctgtacctggccagccactatgagaagctgaagggctcccccgaggataatgagcagaaacagctgtttgtggaacagcacaagcactacctggacgagatcatcgagcagatcagcgagttctccaagagagtgatcctggccgacgctaatctggacaaagtgctgtccgcctacaacaagcaccgggataagcccatcagagagcaggccgagaatatcatccacctgtttaccctgaccaatctgggagcccctgccgccttcaagtactttgacaccaccatcgaccggaagaggtacaccagcaccaaagaggtgctggacgccaccctgatccaccagagcatcaccggcctgtacgagacacggatcgacctgtctcagctgggaggcgacaagcgtcctgctgctactaagaaagctggtcaagctaagaaaaagaaagctagcggcagcggcgccaccaacttcagcctgctgaagcaggccggcgacgtggaggagaaccccggccccatgtctagattagataaaagtaaagtgattaacagcgcattagagctgcttaatgaggtcggaatcgaaggtttaacaacccgtaaactcgcccagaagctaggtgtagagcagcctacattgtattggcatgtaaaaaataagcgggctttgctcgacgccttagccattgagatgttagataggcaccatactcacttttgccctttagaaggggaaagctggcaagattttttacgtaataacgctaaaagttttagatgtgctttactaagtcatcgcgatggagcaaaagtacatttaggtacacggcctacagaaaaacagtatgaaactctcgaaaatcaattagcctttttatgccaacaaggtttttcactagagaatgcattatatgcactcagcgctgtggggcattttactttaggttgcgtattggaagatcaagagcatcaagtcgctaaagaagaaagggaaacacctactactgatagtatgccgccattattacgacaagctatcgaattatttgatcaccaaggtgcagagccagccttcttattcggccttgaattgatcatatgcggattagaaaaacaacttaaatgtgaaagtgggtccgcgtacagccgcgcgcgtacgaaaaacaattacgggtctaccatcgagggcctgctcgatctcccggacgacgacgcccccgaagaggcggggctggcggctccgcgcctgtcctttctccccgcgggacacacgcgcagactgtcgacggcccccccgaccgatgtcagcctgggggacgagctccacttagacggcgaggacgtggcgatggcgcatgccgacgcgctagacgatttcgatctggacatgttgggggacggggattccccgggtccgggatttaccccccacgactccgccccctacggcgctctggatatggccgacttcgagtttgagcagatgtttaccgatgcccttggaattgacgagtacggtgggtagcttaagaaccgctcgaggccggcaaggccggatccagacatgataagatacattgatgagtttggacaaaccacaactagaatgcagtgaaaaaaatgctttatttgtgaaatttgtgatgctattgctttatttgtaaccattataagctgcaataaacaagttaacaacaacaattgcattcattttatgtttcaggttcagggggaggtgtgggaggttttttaaagcaagtaaaacctctacaaatgtggtatggctgattatgatccggctgcctcgcgcgtttcggtgatgacggtgaaaacctctgacacatgcagctcccggagacggtcacagcttgtctgtaagcggatgccgggagcagacaagcccgtcagggcgcgtcagcgggtgttggcgggtgtcggggcgcagccatgaggtcgactctagaggatcgatgccccgccccggacgaactaaacctgactacgacatctctgccccttcttcgcggggcagtgcatgtaatcccttcagttggttggtacaacttgccaactgggccctgttccacatgtgacacggggggggaccaaacacaaaggggttctctgactgtagttgacatccttataaatggatgtgcacatttgccaacactgagtggctttcatcctggagcagactttgcagtctgtggactgcaacacaacattgcctttatgtgtaactcttggctgaagctcttacaccaatgctgggggacatgtacctcccaggggcccaggaagactacgggaggctacaccaacgtcaatcagaggggcctgtgtagctaccgataagcggaccctcaagagggcattagcaatagtgtttataaggcccccttgttaaccctaaacgggtagcatatgcttcccgggtagtagtatatactatccagactaaccctaattcaatagcatatgttacccaacgggaagcatatgctatcgaattagggttagtaaaagggtcctaaggaacagcgatatctcccaccccatgagctgtcacggttttatttacatggggtcaggattccacgagggtagtgaaccattttagtcacaagggcagtggctgaagatcaaggagcgggcagtgaactctcctgaatcttcgcctgcttcttcattctccttcgtttagctaatagaataactgctgagttgtgaacagtaaggtgtatgtgaggtgctcgaaaacaaggtttcaggtgacgcccccagaataaaatttggacggggggttcagtggtggcattgtgctatgacaccaatataaccctcacaaaccccttgggcaataaatactagtgtaggaatgaaacattctgaatatctttaacaatagaaatccatggggtggggacaagccgtaaagactggatgtccatctcacacgaatttatggctatgggcaacacataatcctagtgcaatatgatactggggttattaagatgtgtcccaggcagggaccaagacaggtgaaccatgttgttacactctatttgtaacaaggggaaagagagtggacgccgacagcagcggactccactggttgtctctaacacccccgaaaattaaacggggctccacgccaatggggcccataaacaaagacaagtggccactcttttttttgaaattgtggagtgggggcacgcgtcagcccccacacgccgccctgcggttttggactgtaaaataagggtgtaataacttggctgattgtaaccccgctaaccactgcggtcaaaccacttgcccacaaaaccactaatggcaccccggggaatacctgcataagtaggtgggcgggccaagataggggcgcgattgctgcgatctggaggacaaattacacacacttgcgcctgagcgccaagcacagggttgttggtcctcatattcacgaggtcgctgagagcacggtgggctaatgttgccatgggtagcatatactacccaaatatctggatagcatatgctatcctaatctatatctgggtagcataggctatcctaatctatatctgggtagcatatgctatcctaatctatatctgggtagtatatgctatcctaatttatatctgggtagcataggctatcctaatctatatctgggtagcatatgctatcctaatctatatctgggtagtatatgctatcctaatctgtatccgggtagcatatgctatcctaatagagattagggtagtatatgctatcctaatttatatctgggtagcatatactacccaaatatctggatagcatatgctatcctaatctatatctgggtagcatatgctatcctaatctatatctgggtagcataggctatcctaatctatatctgggtagcatatgctatcctaatctatatctgggtagtatatgctatcctaatttatatctgggtagcataggctatcctaatctatatctgggtagcatatgctatcctaatctatatctgggtagtatatgctatcctaatctgtatccgggtagcatatgctatcctcatgcatatacagtcagcatatgatacccagtagtagagtgggagtgctatcctttgcatatgccgccacctcccaagggggcgtgaattttcgctgcttgtccttttcctgctggttgctcccattcttaggtgaatttaaggaggccaggctaaagccgtcgcatgtctgattgctcaccaggtaaatgtcgctaatgttttccaacgcgagaaggtgttgagcgcggagctgagtgacgtgacaacatgggtatgcccaattgccccatgttgggaggacgaaaatggtgacaagacagatggccagaaatacaccaacagcacgcatgatgtctactggggatttattctttagtgcgggggaatacacggcttttaatacgattgagggcgtctcctaacaagttacatcactcctgcccttcctcaccctcatctccatcacctccttcatctccgtcatctccgtcatcaccctccgcggcagccccttccaccataggtggaaaccagggaggcaaatctactccatcgtcaaagctgcacacagtcaccctgatattgcaggtaggagcgggctttgtcataacaaggtccttaatcgcatccttcaaaacctcagcaaatatatgagtttgtaaaaagaccatgaaataacagacaatggactcccttagcgggccaggttgtgggccgggtccaggggccattccaaaggggagacgactcaatggtgtaagacgacattgtggaatagcaagggcagttcctcgccttaggttgtaaagggaggtcttactacctccatatacgaacacaccggcgacccaagttccttcgtcggtagtcctttctacgtgactcctagccaggagagctcttaaaccttctgcaatgttctcaaatttcgggttggaacctccttgaccacgatgctttccaaaccaccctccttttttgcgcctgcctccatcaccctgaccccggggtccagtgcttgggccttctcctgggtcatctgcggggccctgctctatcgctcccgggggcacgtcaggctcaccatctgggccaccttcttggtggtattcaaaataatcggcttcccctacagggtggaaaaatggccttctacctggagggggcctgcgcggtggagacccggatgatgatgactgactactgggactcctgggcctcttttctccacgtccacgacctctccccctggctctttcacgacttccccccctggctctttcacgtcctctaccccggcggcctccactacctcctcgaccccggcctccactacctcctcgaccccggcctccactgcctcctcgaccccggcctccacctcctgctcctgcccctcctgctcctgcccctcctcctgctcctgcccctcctgcccctcctgctcctgcccctcctgcccctcctgctcctgcccctcctgcccctcctgctcctgcccctcctgcccctcctcctgctcctgcccctcctgcccctcctcctgctcctgcccctcctgcccctcctgctcctgcccctcctgcccctcctgctcctgcccctcctgcccctcctgctcctgcccctcctgctcctgcccctcctgctcctgcccctcctgctcctgcccctcctgcccctcctgcccctcctcctgctcctgcccctcctgctcctgcccctcctgcccctcctgcccctcctgctcctgcccctcctcctgctcctgcccctcctgcccctcctgcccctcctcctgctcctgcccctcctgcccctcctcctgctcctgcccctcctcctgctcctgcccctcctgcccctcctgcccctcctcctgctcctgcccctcctgcccctcctcctgctcctgcccctcctcctgctcctgcccctcctgcccctcctgcccctcctcctgctcctgcccctcctcctgctcctgcccctcctgcccctcctgcccctcctgcccctcctcctgctcctgcccctcctcctgctcctgcccctcctgctcctgcccctcccgctcctgctcctgctcctgttccaccgtgggtccctttgcagccaatgcaacttggacgtttttggggtctccggacaccatctctatgtcttggccctgatcctgagccgcccggggctcctggtcttccgcctcctcgtcctcgtcctcttccccgtcctcgtccatggttatcaccccctcttctttgaggtccactgccgccggagccttctggtccagatgtgtctcccttctctcctaggccatttccaggtcctgtacctggcccctcgtcagacatgattcacactaaaagagatcaatagacatctttattagacgacgctcagtgaatacagggagtgcagactcctgccccctccaacagcccccccaccctcatccccttcatggtcgctgtcagacagatccaggtctgaaaattccccatcctccgaaccatcctcgtcctcatcaccaattactcgcagcccggaaaactcccgctgaacatcctcaagatttgcgtcctgagcctcaagccaggcctcaaattcctcgtccccctttttgctggacggtagggatggggattctcgggacccctcctcttcctcttcaaggtcaccagacagagatgctactggggcaacggaagaaaagctgggtgcggcctgtgaggatcagcttatcgatgataagctgtcaaacatgagaattcttgaagacgaaagggcctcgtgatacgcctatttttataggttaatgtcatgataataatggtttcttagacgtcaggtggcacttttcggggaaatgtgcgcggaacccctatttgtttatttttctaaatacattcaaatatgtatccgctcatgagacaataaccctgataaatgcttcaataatattgaaaaaggaagagtatgagtattcaacatttccgtgtcgcccttattcccttttttgcggcattttgccttcctgtttttgctcacccagaaacgctggtgaaagtaaaagatgctgaagatcagttgggtgcacgagtgggttacatcgaactggatctcaacagcggtaagatccttgagagttttcgccccgaagaacgttttccaatgatgagcacttttaaagttctgctatgtggcgcggtattatcccgtgttgacgccgggcaagagcaactcggtcgccgcatacactattctcagaatgacttggttgagtactcaccagtcacagaaaagcatcttacggatggcatgacagtaagagaattatgcagtgctgccataaccatgagtgataacactgcggccaacttacttctgacaacgatcggaggaccgaaggagctaaccgcttttttgcacaacatgggggatcatgtaactcgccttgatcgttgggaaccggagctgaatgaagccataccaaacgacgagcgtgacaccacgatgcctgcagcaatggcaacaacgttgcgcaaactattaactggcgaactacttactctagcttcccggcaacaattaatagactggatggaggcggataaagttgcaggaccacttctgcgctcggcccttccggctggctggtttattgctgataaatctggagccggtgagcgtgggtctcgcggtatcattgcagcactggggccagatggtaagccctcccgtatcgtagttatctacacgacggggagtcaggcaactatggatgaacgaaatagacagatcgctgagataggtgcctcactgattaagcattggtaactgtcagaccaagtttactcatatatactttagattgatttaaaacttcatttttaatttaaaaggatctaggtgaagatcctttttgataatctcatgaccaaaatcccttaacgtgagttttcgttccactgagcgtcagaccccgtagaaaagatcaaaggatcttcttgagatcctttttttctgcgcgtaatctgctgcttgcaaacaaaaaaaccaccgctaccagcggtggtttgtttgccggatcaagagctaccaactctttttccgaaggtaactggcttcagcagagcgcagataccaaatactgtccttctagtgtagccgtagttaggccaccacttcaagaactctgtagcaccgcctacatacctcgctctgctaatcctgttaccagtggctgctgccagtggcgataagtcgtgtcttaccgggttggactcaagacgatagttaccggataaggcgcagcggtcgggctgaacggggggttcgtgcacacagcccagcttggagcgaacgacctacaccgaactgagatacctacagcgtgagctatgagaaagcgccacgcttcccgaagggagaaaggcggacaggtatccggtaagcggcagggtcggaacaggagagcgcacgagggagcttccagggggaaacgcctggtatctttatagtcctgtcgggtttcgccacctctgacttgagcgtcgatttttgtgatgctcgtcaggggggcggagcctatggaaaaacgccagcaacgcggcctttttacggttcctggccttttgctggccttgaagctgtccctgatggtcgtcatctacctgcctggacagcatggcctgcaacgcgggcatcccgatgccgccggaagcgagaagaatcataatggggaaggccatccagcctcgcgtcgaactagatgatccggctgtggaatgtgtgtcagttagggtgtggaaagtccccaggctccccagcaggcagaagtatgcaaagcatg

**Fig. S9. KO plasmid sequence (**human U6 promoter colored in red, gRNA scaffold colored in green, BspQI restriction sites underlined, EF1a promoter colored in blue, spCas9 colored in pink, tTA colored in purple, EBNA1/Orip sequence colored in grey.)

ttagggtgtggaaagtccccaggctccccagcaggcagaagtatgcaaagcatgcgagtttactccctatcagtgatagagaacgtatgtcgagtttactccctatcagtgatagagaacgatgtcgagtttactccctatcagtgatagagaacgtatgtcgagtttactccctatcagtgatagagaacgtatgtcgagtttactccctatcagtgatagagaacgtatgtcgagtttatccctatcagtgatagagaacgtatgtcgagtttactccctatcagtgatagagaacgtatgtcgaggtaggcgtgtacggtgggaggcctatataagcagagctcgtttagtgaaccgtcagatcgcctggagaattcgagctcggtacccggggatcctctagtcagctgacgcgtgctagcgcggccgcatcgataagcttgtcgacgatatctctagagcgatcgctgaccggtgagggcagaggaagtcttctaacatgcggtgacgtggaggagaatcccggcccttccggaatggagagcgacgagagcggcctgcccgccatggagatcgagtgccgcatcaccggcaccctgaacggcgtggagttcgagctggtgggcggcggagagggcacccccaagcagggccgcatgaccaacaagatgaaaagcaccaaaggcgccctgaccttcagcccctacctgctgagccacgtgatgggctacggcttctaccacttcggcacctaccccagcggctacgagaaccccttcctgcacgccatcaacaacggcggctacaccaacacccgcatcgagaagtacgaggacggcggcgtgctgcacgtgagcttcagctaccgctacgaggccggccgcgtgatcggcgacttcaaggtggtgggcaccggcttccccgaggacagcgtgatcttcaccgacaagatcatccgcagcaacgccaccgtggagcacctgcaccccatgggcgataacgtgctggtgggcagcttcgcccgcaccttcagcctgcgcgacggcggctactacagcttcgtggtggacagccacatgcacttcaagagcgccatccaccccagcatcctgcagaacgggggccccatgttcgccttccgccgcgtggaggagctgcacagcaacaccgagctgggcatcgtggagtaccagcacgccttcaagacccccatcgccttcgccagatcccgcgctcagtcgtccaattctgccgtggacggcaccgccggacccggctccaccggatctcgcCttaagggcagcggcgccaccaacttcagcctgctgaagcaggccggcgacgtggaggagaaccccggccccatgaccgagtacaagcccacggtgcgcctcgccacccgcgacgacgtccccagggccgtacgcaccctcgccgccgcgttcgccgactaccccgccacgcgccacaccgtcgatccggaccgccacatcgagcgggtcaccgagctgcaagaactcttcctcacgcgcgtcgggctcgacatcggcaaggtgtgggtcgcggacgacggcgccgcggtggcggtctggaccacgccggagagcgtcgaagcgggggcggtgttcgccgagatcggcccgcgcatggccgagttgagcggttcccggctggccgcgcagcaacagatggaaggcctcctggcgccgcaccggcccaaggagcccgcgtggttcctggccaccgtcggcgtctcgcccgaccaccagggcaagggtctgggcagcgccgtcgtgctccccggagtggaggcggccgagcgcgccggggtgcccgccttcctggagacctccgcgccccgcaacctccccttctacgagcggctcggcttcaccgtcaccgccgacgtcgaggtgcccgaaggaccgcgcacctggtgcatgacccgcaagcccggtgccggatccatgcccacgctactgcgggtttatatagacggtcctcacgggatggggaaaaccaccaccacgcaactgctggtggccctgggttcgcgcgacgatatcgtctacgtacccgagccgatgacttactggcaggtgctgggggcttccgagacaatcgcgaacatctacaccacacaacaccgcctcgaccagggtgagatatcggccggggacgcggcggtggtaatgacaagcgcccagataacaatgggcatgccttatgccgtgaccgacgccgttctggctcctcatatcgggggggaggctgggagctcacatgccccgcccccggccctcaccctcatcttcgaccgccatcccatcgccgccctcctgtgctacccggccgcgcgataccttatgggcagcatgaccccccaggccgtgctggcgttcgtggccctcatcccgccgaccttgcccggcacaaacatcgtgttgggggcccttccggaggacagacacatcgaccgcctggccaaacgccagcgccccggcgagcggcttgacctggctatgctggccgcgattcgccgcgtttacgggctgcttgccaatacggtgcggtatctgcagggcggcgggtcgtggcgggaggattggggacagctttcggggacggccgtgccgccccagggtgccgagccccagagcaacgcgggcccacgaccccatatcggggacacgttatttaccctgtttcgggcccccgagttgctggcccccaacggcgacctgtacaacgtgtttgcctgggccttggacgtcttggccaaacgcctccgtcccatgcacgtctttatcctggattacgaccaatcgcccgccggctgccgggacgccctgctgcaacttacctccgggatggtccagacccacgtcaccacccccggctccataccgacgatctgcgacctggcgcgcacgtttgcccgggagatgggggaggctaactgactcgaggccggcaaggccggatccagacatgataagatacattgatgagtttggacaaaccacaactagaatgcagtgaaaaaaatgctttatttgtgaaatttgtgatgctattgctttatttgtaaccattataagctgcaataaacaagttaacaacaacaattgcattcattttatgtttcaggttcagggggaggtgtgggaggttttttaaagcaagtaaaacctctacaaatgtggtatggctgattatgatccggctgcctcgcgcgtttcggtgatgacggtgaaaacctctgacacatgcagctcccggagacggtcacagcttgtctgtaagcggatgccgggagcagacaagcccgtcagggcgcgtcagcgggtgttggcgggtgtcggggcgcagccatgaggtcgactctagaggatcgatgccccgccccggacgaactaaacctgactacgacatctctgccccttcttcgcggggcagtgcatgtaatcccttcagttggttggtacaacttgccaactgggccctgttccacatgtgacacggggggggaccaaacacaaaggggttctctgactgtagttgacatccttataaatggatgtgcacatttgccaacactgagtggctttcatcctggagcagactttgcagtctgtggactgcaacacaacattgcctttatgtgtaactcttggctgaagctcttacaccaatgctgggggacatgtacctcccaggggcccaggaagactacgggaggctacaccaacgtcaatcagaggggcctgtgtagctaccgataagcggaccctcaagagggcattagcaatagtgtttataaggcccccttgttaaccctaaacgggtagcatatgcttcccgggtagtagtatatactatccagactaaccctaattcaatagcatatgttacccaacgggaagcatatgctatcgaattagggttagtaaaagggtcctaaggaacagcgatatctcccaccccatgagctgtcacggttttatttacatggggtcaggattccacgagggtagtgaaccattttagtcacaagggcagtggctgaagatcaaggagcgggcagtgaactctcctgaatcttcgcctgcttcttcattctccttcgtttagctaatagaataactgctgagttgtgaacagtaaggtgtatgtgaggtgctcgaaaacaaggtttcaggtgacgcccccagaataaaatttggacggggggttcagtggtggcattgtgctatgacaccaatataaccctcacaaaccccttgggcaataaatactagtgtaggaatgaaacattctgaatatctttaacaatagaaatccatggggtggggacaagccgtaaagactggatgtccatctcacacgaatttatggctatgggcaacacataatcctagtgcaatatgatactggggttattaagatgtgtcccaggcagggaccaagacaggtgaaccatgttgttacactctatttgtaacaaggggaaagagagtggacgccgacagcagcggactccactggttgtctctaacacccccgaaaattaaacggggctccacgccaatggggcccataaacaaagacaagtggccactcttttttttgaaattgtggagtgggggcacgcgtcagcccccacacgccgccctgcggttttggactgtaaaataagggtgtaataacttggctgattgtaaccccgctaaccactgcggtcaaaccacttgcccacaaaaccactaatggcaccccggggaatacctgcataagtaggtgggcgggccaagataggggcgcgattgctgcgatctggaggacaaattacacacacttgcgcctgagcgccaagcacagggttgttggtcctcatattcacgaggtcgctgagagcacggtgggctaatgttgccatgggtagcatatactacccaaatatctggatagcatatgctatcctaatctatatctgggtagcataggctatcctaatctatatctgggtagcatatgctatcctaatctatatctgggtagtatatgctatcctaatttatatctgggtagcataggctatcctaatctatatctgggtagcatatgctatcctaatctatatctgggtagtatatgctatcctaatctgtatccgggtagcatatgctatcctaatagagattagggtagtatatgctatcctaatttatatctgggtagcatatactacccaaatatctggatagcatatgctatcctaatctatatctgggtagcatatgctatcctaatctatatctgggtagcataggctatcctaatctatatctgggtagcatatgctatcctaatctatatctgggtagtatatgctatcctaatttatatctgggtagcataggctatcctaatctatatctgggtagcatatgctatcctaatctatatctgggtagtatatgctatcctaatctgtatccgggtagcatatgctatcctcatgcatatacagtcagcatatgatacccagtagtagagtgggagtgctatcctttgcatatgccgccacctcccaagggggcgtgaattttcgctgcttgtccttttcctgctggttgctcccattcttaggtgaatttaaggaggccaggctaaagccgtcgcatgtctgattgctcaccaggtaaatgtcgctaatgttttccaacgcgagaaggtggaattccaggtggcacttttcggggaaatgtgcgcggaacccctatttgtttatttttctaaatacattcaaatatgtatccgctcatgagacaataaccctgataaatgcttcaataatattgaaaaaggaagagtatgagtattcaacatttccgtgtcgcccttattcccttttttgcggcattttgccttcctgtttttgctcacccagaaacgctggtgaaagtaaaagatgctgaagatcagttgggtgcacgagtgggttacatcgaactggatctcaacagcggtaagatccttgagagttttcgccccgaagaacgttttccaatgatgagcacttttaaagttctgctatgtggcgcggtattatcccgtgttgacgccgggcaagagcaactcggtcgccgcatacactattctcagaatgacttggttgagtactcaccagtcacagaaaagcatcttacggatggcatgacagtaagagaattatgcagtgctgccataaccatgagtgataacactgcggccaacttacttctgacaacgatcggaggaccgaaggagctaaccgcttttttgcacaacatgggggatcatgtaactcgccttgatcgttgggaaccggagctgaatgaagccataccaaacgacgagcgtgacaccacgatgcctgcagcaatggcaacaacgttgcgcaaactattaactggcgaactacttactctagcttcccggcaacaattaatagactggatggaggcggataaagttgcaggaccacttctgcgctcggcccttccggctggctggtttattgctgataaatctggagccggtgagcgtgggtctcgcggtatcattgcagcactggggccagatggtaagccctcccgtatcgtagttatctacacgacggggagtcaggcaactatggatgaacgaaatagacagatcgctgagataggtgcctcactgattaagcattggtaactgtcagaccaagtttactcatatatactttagattgatttaaaacttcatttttaatttaaaaggatctaggtgaagatcctttttgataatctcatgaccaaaatcccttaacgtgagttttcgttccactgagcgtcagaccccgtagaaaagatcaaaggatcttcttgagatcctttttttctgcgcgtaatctgctgcttgcaaacaaaaaaaccaccgctaccagcggtggtttgtttgccggatcaagagctaccaactctttttccgaaggtaactggcttcagcagagcgcagataccaaatactgtccttctagtgtagccgtagttaggccaccacttcaagaactctgtagcaccgcctacatacctcgctctgctaatcctgttaccagtggctgctgccagtggcgataagtcgtgtcttaccgggttggactcaagacgatagttaccggataaggcgcagcggtcgggctgaacggggggttcgtgcacacagcccagcttggagcgaacgacctacaccgaactgagatacctacagcgtgagctatgagaaagcgccacgcttcccgaagggagaaaggcggacaggtatccggtaagcggcagggtcggaacaggagagcgcacgagggagcttccagggggaaacgcctggtatctttatagtcctgtcgggtttcgccacctctgacttgagcgtcgatttttgtgatgctcgtcaggggggcggagcctatggaaaaacgccagcaacgcggcctttttacggttcctggccttttgctggccttgaagctgtccctgatggtcgtcatctacctgcctggacagcatggcctgcaacgcgggcatcccgatgccgccggaagcgagaagaatcataatggggaaggccatccagcctcgcgtcgaactagatgatccggctgtggaatgtgtgtcag

**Fig. S10. Rsecue plasmid sequence (**TRE promoter colored in orange, KpnI and AsisI restriction sites underlined, GFP colored in green, puromycin resistence gene colored in blue, P2A colored in pink, Orip sequence colored in grey.)

cgagtttactccctatcagtgatagagaacgtatgtcgagtttactccctatcagtgatagagaacgatgtcgagtttactccctatcagtgatagagaacgtatgtcgagtttactccctatcagtgatagagaacgtatgtcgagtttactccctatcagtgatagagaacgtatgtcgagtttatccctatcagtgatagagaacgtatgtcgagtttactccctatcagtgatagagaacgtatgtcgaggtaggcgtgtacggtgggaggcctatataagcagagctcgtttagtgaaccgtcagatcgcctggagaattcgagctcggtacccggggatcctctagtcagctgacgcgtgctagcgcggccgcatcgataagcttgtcgacgatatctctagagcgatcgcgagggcagaggaagtcttctaacatgcggtgacgtggaggagaatcccggccctatggacaacaccgaggacgtcatcaaggagttcatgcagttcaaggtgcgcatggagggctccgtgaacggccactacttcgagatcgagggcgagggcgagggcaagccctacgagggcacccagaccgccaagctgcaggtgaccaagggcggccccctgcccttcgcctgggacatcctgtccccccagttccagtacggctccaaggcctacgtgaagcaccccgccgacatccccgactacatgaagctgtccttccccgagggcttcacctgggagcgctccatgaacttcgaggacggcggcgtggtggaggtgcagcaggactcctccctgcaggacggcaccttcatctacaaggtgaagttcaagggcgtgaacttccccgccgacggccccgtaatgcagaagaagactgccggctgggagccctccaccgagaagctgtacccccaggacggcgtgctgaagggcgagatctcccacgccctgaagctgaaggacggcggccactacacctgcgacttcaagaccgtgtacaaggccaagaagcccgtgcagctgcccggcaaccactacgtggactccaagctggacatcaccaaccacaacgaggactacaccgtggtggagcagtacgagcacgccgaggcccgccactccggctcccaggtaagtatcaaggagagcgcttcttgccgcctttcactcttgcgtttctgataggcacctattggtcttactgacttaagctgtggaatgtgtgtcagttagggtgtggaaagtccccaggctccccaggcaggcagaagtatgcaaagcatgcatctcaattagtcagcaaccaggtgtggaaagtccccaggctccccagcaggcagaagtatgcaaagcatgcatctcaattagtcagcaaccatagtcccgcccctaactccgcccatcccgcccctaactccgcccagttccgcccattctccgccccatggctgactaattttttttatttatgcagaggccgaggccgcctctgcctctgagctattccagaagtagtgaggaggcttttttggaggcctaggcttttgcaaaaagctcccgggagcttgtatatccattttcggatctgatcagcacgtgttgacaattaatcatcggcatagtatatcggcatagtataatacgacaaggtgaggaactaaaccatggccaagttgaccagtgccgttccggtgctcaccgcgcgcgacgtcgccggagcggtcgagttctggaccgaccggctcgggttctcccgggacttcgtggaggacgacttcgccggtgtggtccgggacgacgtgaccctgttcatcagcgcggtccaggaccaggtggtgccggacaacaccctggcctgggtgtgggtgcgcggcctggacgagctgtacgccgagtggtcggaggtcgtgtccacgaacttccgggacgcctccgggccggccatgaccgagatcggcgagcagccgtgggggcgggagttcgccctgcgcgacccggccggcaactgcgtgcacttcgtggccgaggagcaggactgacacgtgctacgagatttcgattcctcgaggccggcaaggccggatccagacatgataagatacattgatgagtttggacaaaccacaactagaatgcagtgaaaaaaatgctttatttgtgaaatttgtgatgctattgctttatttgtaaccattataagctgcaataaacaagttaacaacaacaattgcattcattttatgtttcaggttcagggggaggtgtgggaggttttttaaagcaagtaaaacctctacaaatgtggtatggctgattatgatccggctgcctcgcgcgtttcggtgatgacggtgaaaacctctgacacatgcagctcccggagacggtcacagcttgtctgtaagcggatgccgggagcagacaagcccgtcagggcgcgtcagcgggtgttggcgggtgtcggggcgcagccatgaggtcgactctagaggatcgatgccccgccccggacgaactaaacctgactacgacatctctgccccttcttcgcggggcagtgcatgtaatcccttcagttggttggtacaacttgccaactgggccctgttccacatgtgacacggggggggaccaaacacaaaggggttctctgactgtagttgacatccttataaatggatgtgcacatttgccaacactgagtggctttcatcctggagcagactttgcagtctgtggactgcaacacaacattgcctttatgtgtaactcttggctgaagctcttacaccaatgctgggggacatgtacctcccaggggcccaggaagactacgggaggctacaccaacgtcaatcagaggggcctgtgtagctaccgataagcggaccctcaagagggcattagcaatagtgtttataaggcccccttgttaaccctaaacgggtagcatatgcttcccgggtagtagtatatactatccagactaaccctaattcaatagcatatgttacccaacgggaagcatatgctatcgaattagggttagtaaaagggtcctaaggaacagcgatatctcccaccccatgagctgtcacggttttatttacatggggtcaggattccacgagggtagtgaaccattttagtcacaagggcagtggctgaagatcaaggagcgggcagtgaactctcctgaatcttcgcctgcttcttcattctccttcgtttagctaatagaataactgctgagttgtgaacagtaaggtgtatgtgaggtgctcgaaaacaaggtttcaggtgacgcccccagaataaaatttggacggggggttcagtggtggcattgtgctatgacaccaatataaccctcacaaaccccttgggcaataaatactagtgtaggaatgaaacattctgaatatctttaacaatagaaatccatggggtggggacaagccgtaaagactggatgtccatctcacacgaatttatggctatgggcaacacataatcctagtgcaatatgatactggggttattaagatgtgtcccaggcagggaccaagacaggtgaaccatgttgttacactctatttgtaacaaggggaaagagagtggacgccgacagcagcggactccactggttgtctctaacacccccgaaaattaaacggggctccacgccaatggggcccataaacaaagacaagtggccactcttttttttgaaattgtggagtgggggcacgcgtcagcccccacacgccgccctgcggttttggactgtaaaataagggtgtaataacttggctgattgtaaccccgctaaccactgcggtcaaaccacttgcccacaaaaccactaatggcaccccggggaatacctgcataagtaggtgggcgggccaagataggggcgcgattgctgcgatctggaggacaaattacacacacttgcgcctgagcgccaagcacagggttgttggtcctcatattcacgaggtcgctgagagcacggtgggctaatgttgccatgggtagcatatactacccaaatatctggatagcatatgctatcctaatctatatctgggtagcataggctatcctaatctatatctgggtagcatatgctatcctaatctatatctgggtagtatatgctatcctaatttatatctgggtagcataggctatcctaatctatatctgggtagcatatgctatcctaatctatatctgggtagtatatgctatcctaatctgtatccgggtagcatatgctatcctaatagagattagggtagtatatgctatcctaatttatatctgggtagcatatactacccaaatatctggatagcatatgctatcctaatctatatctgggtagcatatgctatcctaatctatatctgggtagcataggctatcctaatctatatctgggtagcatatgctatcctaatctatatctgggtagtatatgctatcctaatttatatctgggtagcataggctatcctaatctatatctgggtagcatatgctatcctaatctatatctgggtagtatatgctatcctaatctgtatccgggtagcatatgctatcctcatgcatatacagtcagcatatgatacccagtagtagagtgggagtgctatcctttgcatatgccgccacctcccaagggggcgtgaattttcgctgcttgtccttttcctgctggttgctcccattcttaggtgaatttaaggaggccaggctaaagccgtcgcatgtctgattgctcaccaggtaaatgtcgctaatgttttccaacgcgagaaggtggaattccaggtggcacttttcggggaaatgtgcgcggaacccctatttgtttatttttctaaatacattcaaatatgtatccgctcatgagacaataaccctgataaatgcttcaataatattgaaaaaggaagagttaactgtcagaccaagtttactcatatatactttagattgatttaaaacttcatttttaatttaaaaggatctaggtgaagatcctttttgataatctcatgaccaaaatcccttaacgtgagttttcgttccactgagcgtcagaccccgtagaaaagatcaaaggatcttcttgagatcctttttttctgcgcgtaatctgctgcttgcaaacaaaaaaaccaccgctaccagcggtggtttgtttgccggatcaagagctaccaactctttttccgaaggtaactggcttcagcagagcgcagataccaaatactgtccttctagtgtagccgtagttaggccaccacttcaagaactctgtagcaccgcctacatacctcgctctgctaatcctgttaccagtggctgctgccagtggcgataagtcgtgtcttaccgggttggactcaagacgatagttaccggataaggcgcagcggtcgggctgaacggggggttcgtgcacacagcccagcttggagcgaacgacctacaccgaactgagatacctacagcgtgagctatgagaaagcgccacgcttcccgaagggagaaaggcggacaggtatccggtaagcggcagggtcggaacaggagagcgcacgagggagcttccagggggaaacgcctggtatctttatagtcctgtcgggtttcgccacctctgacttgagcgtcgatttttgtgatgctcgtcaggggggcggagcctatggaaaaacgccagcaacgcggcctttttacggttcctggccttttgctggccttgaagctgtccctgatggtcgtcatctacctgcctggacagcatggcctgcaacgcgggcatcccgatgccgccggaagcgagaagaatcataatggggaaggccatccagcctcgcgtcgaactagatgatccggctgtggaatgtgtgtcagttagggtgtggaaagtccccaggctccccagcaggcagaagtatgcaaagcatg

**Fig. S11. Rsecue2 plasmid sequence (**TRE promoter colored in orange, KpnI and AsisI restriction sites underlined, RFP colored in red, zeocin resistence gene colored in blue, Orip sequence colored in grey.)

**Table S1. Primers and oligonucleotides**

| **Name** | **Sequence** | **Description** |
| --- | --- | --- |
| T7-DNMT1-F1 | CCGTAGGCTCCATCTCTGAA | Primers for amplification of the engogenous targets |
| T7-DNMT1-F2 | ACCTGAAACGACTTCCAACC |  |
| T7-DNMT1-R1 | ACCCGTTTACCTGAGGAAGG |  |
| T7-DNMT1-R2 | CCTCCACAGACAGAGGGAAG |  |
| T7-HDAC3-F2 | GACCTCCCTCCTCCCTGATT |  |
| T7-HDAC3-R2 | GAGGAGGGGACACCTGAGAT |  |
| T7-PRAP1-F2 | GTGTTTCTAGGTCGTGGCGT |  |
| T7-PRAP1-R2 | CGGGCTTTTCCTGCAACATC |  |
| q-p-DNMT1-F | ATGTGGGATCCAGCCGCGCAA | Primers for amplification of the specifically exogenous cDNA |
| q-p-DNMT1-R | CATCTCCACGTCTCCCTTCT |  |
| q-p-HDAC3-F | GATCGCGTGATGACTGTCAG |  |
| q-p-HDAC3-R | GTTGATAACCGGCTGGAAAA |  |
| q-p-PARP1-F | CGCGTGGAATATGCTAAATCCG |  |
| q-p-PARP1-R | TCAGAGAACCCATCCACCTC |  |
| q-GAPDH-F | GAAGGTGAAGGTCGGAGTC | Primers for amplification of the internal control gene GAPDH |
| q-GAPDH-R | GAAGATGGTGATGGGATTTC |  |
| sgRNA-DNMT1-F | CCGGATGTGGGACCCTGCGGCCC | Primers used to anneal to form oligonucleotide duplexes |
| sgRNA-DNMT1-R | AACGGGCCGCAGGGTCCCACATC |  |
| sgRNA-HDAC3-F | CCGGGAAGGACACCGTCATGACC |  |
| sgRNA-HDAC3-R | AACGGTCATGACGGTGTCCTTCC |  |
| sgRNA-PARP1-F | CCGCGAGTCGAGTACGCCAAGAG |  |
| sgRNA-PARP1-R | AACCTCTTGGCGTACTCGACTCG |  |
| His97Arg-F | AACGGTGCCCGGGCTTACAACCGGGAAGTGAA | Primers used to introduce point mutations in exogenous cDNA sequences |
| His97Arg-R | TTGTAAGCCCGG GCACCGTTCTCCAAGGACAA |  |
| Pro507Leu-F | TGATGGATCTCAGTCCCGAGTATGCGCCCA |  |
| Pro507Leu-R | CTCGGGACTGAGATCCATCAGAATGTATTCGGC |  |
| Tyr511Cys-F | CAGTCCGGAGTGTGCGCCCATATTTGGGCTGA |  |
| Tyr511Cys-R | GGCGCACACTCCGGACTGGGATCCATCAGAATG |  |
| Ala570Val-F | TGCGACACGTGCAGTTTGTGGTGGAGCAG |  |
| Ala570Val-R | ACAAACTGCACGTGTCGCAGGAGGGAGT |  |
| Gibson-DNMT1-R | CTTCCTCTGCCCTCGCGATCGCA GTCCTTAGCAGCTTCCTCCTCC |  |
| Gibson-DNMT1-F1 | GAGAATTCGAGCTCGGTACCGCCACCATGC |  |
| His97Arg-xmaI-F | GGCGGCTCAAAGATTTGGAAAGA |  |
| His97Arg-xmaI-R | CAGGAAGCGGTCTAGCAACT |  |
| A570V-PmelI-F | GAGCAATTCCGACTCGACCT |  |
| A570V-PmelI-R | CATATTGGGACACCTCCGCT |  |
| DNMT1-OT1-F | ACTCTGGACAGCCCACAATG | Primers for amplification of the engogenous off targets |
| DNMT1-OT1-R | GGTTGCTGGGCATTTCCTTG |  |
| DNMT1-OT2-F | CACCCATCAGGAAGCTCAGG |  |
| DNMT1-OT2-R | TGCTGGCTAATCCCCTTTGG |  |
| DNMT1-OT3-F | CACATAAAATTGCCTGCGGCT |  |
| DNMT1-OT3-R | GGGTTCACTTCCTCCTCAGC |  |
| DNMT1-OT3-F2 | GGCTTGTCTGGCTCTCTCAT |  |
| DNMT1-OT3-R2 | CATTGTTACCCCTCCTGCCT |  |
| DNMT1-OT4-F | CACTGTAGGGCTTTCTGGGG |  |
| DNMT1-OT4-R | CTCTTGGCACATCGTGACCT |  |
| DNMT1-OT4-F2 | AGTCAGGGTCCATGTGCAAG |  |
| DNMT1-OT4-R2 | ATGACTTCTGAGCCTGGGAG |  |
| DNMT1-OT5-F | CCAAAGGACAACCCGAAGGA |  |
| DNMT1-OT5-R | AGCTGTGGGTCAGAGAGAGT |  |
| DNMT1-OT5-F2 | CAGCTGGGTGAGAGGAGG |  |
| DNMT1-OT5-R2 | TCAACCCACGTGCTCTGTAA |  |
| HDAC3-OT1-F | AGCCAGTTTCCACGTCTCTG |  |
| HDAC3-OT1-R | AGTGAGTCAGGAGACCCCTC |  |
| HDAC3-OT2-F | GTCTCTGCCTTTCCACTCCC |  |
| HDAC3-OT2-R | CTGGCTCCATGCAATGCTTC |  |
| HDAC3-OT2-F2 | CATGGACTCAGGAGGGGAAC |  |
| HDAC3-OT2-R2 | CCAGGGTACATGTGCAGGA |  |
| HDAC3-OT3-F | ACCTTGCCCCTAATGAAGGC |  |
| HDAC3-OT3-R | GTAAGGGCATGAGGGCAAGT |  |
| HDAC3-OT4-F1 | TTGGGACGCTAGTAGAGGGT |  |
| HDAC3-OT4-R1 | AGGCATCACACAAAGCTGGA |  |
| HDAC3-OT4-F2 | GTCTCCTGGAGCTCAAGTGA |  |
| HDAC3-OT4-R2 | CAGGGATGGAGGAGCAGATT |  |
| HDAC3-OT5-F | GCCACACATAGGCCAGAACT |  |
| HDAC3-OT5-R | ACACCTCGCTCCATCTACCT |  |
